# A Palynological Atlas of the Amazon *canga* Vegetation

**DOI:** 10.3390/plants14091319

**Published:** 2025-04-27

**Authors:** Luiza de Araújo Romeiro, Edilson Freitas da Silva, Luiza Santos Reis, Léa Maria Medeiros Carreira, Tarcísio Magevski Rodrigues, Delmo Fonseca da Silva, Tereza Cristina Giannini, Markus Gastauer, Pedro Walfir Martins e Souza-Filho, Lourival Tyski, José Tasso Felix Guimarães

**Affiliations:** 1 Vale Institute of Technology, Rua Boaventura da Silva 955, Nazaré, Belém 66055-090, PA, Brazil; luizaromeiro84@gmail.com (L.d.A.R.); freitasdasilva20@yahoo.com.br (E.F.d.S.); luiza_sreis@yahoo.com.br (L.S.R.); tereza.giannini@itv.org (T.C.G.); markus.gastauer@itv.org (M.G.); 2 Departamento de Botânica, Museu Paraense Emílio Goeldi, Terra Firme, Belém 66040-170, PA, Brazil; lea@museu-goeldi.br; 3 WSP Brasil, Rua Antônio de Albuquerque 194, 7 andar, Funcionários, Belo Horizonte 30112-010, MG, Brazil; tarcisiomr@gmail.com; 4 Independent Researcher, Rua Bejamin 454–Betânia, Parauapebas 68515-000, PA, Brazil; 5 Geosciences Institute, Universidade Federal do Pará, Belém 66075-110, PA, Brazil; pedropwm@gmail.com; 6 Gerência de Estudos Técnicos, Estrada Raymundo Mascarenhas, S/N Carajás, Parauapebas 68516-000, PA, Brazil; lourival.tyski@vale.com

**Keywords:** *canga* vegetation, Carajás, palynology, Amazonia, pollen, spores

## Abstract

*cangas* are iron-rich outcrops where rupestrian fields develop in the Carajás Mountain Range (CMR). *canga* formations are ancient ecosystems characterized by high levels of endemic and threatened plant species that thrive on iron-rich substrates in the southeastern Amazon uplands. The recent taxonomic validation of these species enables more accurate distribution modeling across past, present, and future time scales. This work presents a comprehensive palynological database for the Amazon *canga* vegetation, resulting from extensive field and herbarium surveys, as well as the compilation and taxonomic validation of species in the Carajás Mountain Range (CMR). This atlas includes 204 plant species: 10 ferns and lycophytes, 62 monocots, and 132 eudicots and magnoliids (mainly herbs, lianas, and trees). Most flowering plants are pollinated by bees, with secondary pollination by other insects and wind. The taxa co-occur in two geoenvironments: (1) forested slopes and caves over plinthosols and ferralsols and (2) slopes with *canga* vegetation over plinthosols. Seventeen species are potential domesticates used by Indigenous peoples. This highlights *canga* vegetation as a unique and diverse ecosystem with various survival strategies, emphasizing the need for precise habitat definitions in paleoenvironmental and paleoclimate reconstructions. This atlas provides a valuable reference for palynological studies, enhancing the vegetation reconstruction, climate history analysis, pre-Columbian influences on vegetation patterns, and ecological monitoring.

## 1. Introduction

Amazonia is often cited as having the most diverse flora on the planet [1,2,3], which includes mountain areas with a small surface area but with a fascinating endemism [4]. However, these regions are seriously threatened by extensive land use and land cover changes that have occurred over the past few decades [5,6]. In fact, the flora of these areas presents a great variety of endangered, endemic, and rare taxa in conjunction with a species diversity that represents a highly relevant reserve of biodiversity for the global prioritization of conservation efforts [7], defined as Key Biodiversity Areas—KBAs [8].

From the 149 KBAs identified within the Amazon Rainforest biome, the Carajás Mountain Range—CMR, in southeastern Amazonia, presents 10 rare species [9]. The CMR hosts one of the largest mineral provinces in the world [10], with mountain tops covered by herbaceous and shrubby montane savanna (*canga* vegetation) and associated with lateritic outcrops at 600–800 m altitudes, which are surrounded by rainforests on mountain slopes [11,12]. *The canga* vegetation occupied an area of 144.2 km^2^ in the CMR in 1973, before the implementation of the Carajás N4-N5 open-pit mines, and up to 2016, 22 km^2^ of *canga* vegetation was suppressed in response to iron ore exploitation [13]. The *canga* formations in Brazil are considered ancient ecosystems, characterized by a rich flora associated with the weathering of the iron-rich substrate [14,15,16,17]. In both countries, such areas provide unique island-like environments with high levels of species turnover between different sites, high levels of endemism, and rare geographically restricted species [16,17,18,19,20].

Based on efforts to collect, compile, and taxonomically validate the plant species from 2015 to 2018, during the Flora of Carajás project, certified lists of 856 species of seed plants and 186 species of Ferns and Lycophytes were made available online [16,17]. From these lists, three monotypic genera and thirty-eight species were reported as endemic [4]. This presents a significant opportunity to create a comprehensive palynological database for the Amazon *canga* vegetation. A pollen database with a precise morphological description favored the development of (1) palynotaxonomy (Convolvulaceae [21], Poaceae [22]), (2) melissopalynology (honey pollen samples of *Melipona seminigra pernigra* Moure & Kerr, 1950 [23]), (3) pollen loads from bee specimens deposited in biological collections [24], and (4) paleoecology (lake sediments [25,26,27,28,29,30]). Pollen from bat guano found in the Carajás caves improves our understanding of the area’s ecology and the complex interactions between plants and bats that developed while bats searched for and collected resources [31,32]. However, significant effort is needed for identification at the lowest taxonomic levels. This is highly necessary to (1) develop accurate pollen interaction networks based on floral visitors, (2) precisely describe the paleobiogeographic patterns of *canga* vegetation, and (3) model the Quaternary paleohydrology and paleoclimate based on palynological data. Consequently, this work aims to create an illustrated and descriptive pollen atlas, serving as a valuable tool for researchers worldwide who wish to further explore the palynology of the Amazon *canga* vegetation.

### Study Area

**Figure 1:**
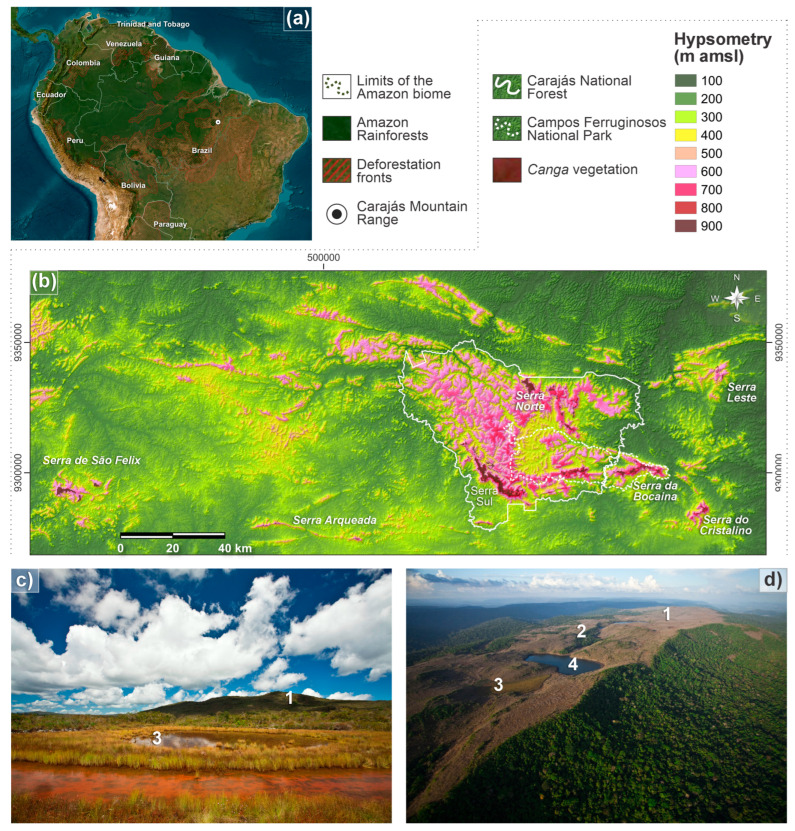
The study site location. (**a**) The location within South America, highlighting the deforestation fronts in the Amazon Biome from 2014 to 2020 [36], which have exerted greater pressure on the southeastern region; (**b**) the distribution of *canga* outcrops along the Carajás Mountain Range—CMR [37], particularly within law-protected areas, such as the Carajás National Forest and the Campos Ferruginosos National Park. The hypsometry in meters above the mean sea-level (amsl); (**c**,**d**), the front view and aerial photographs, respectively, of the *canga* geoenvironments (by João M. Rosa). 1: Slopes with rupestrian *canga* vegetation over plinthosols; 2: forested slopes and caves over plinthosols and ferralsols; 3: poorly drained depressions and levels covered by grasslands over plinthosols and histosols; and 4: doliniform lakes with organic mud sediment at the bottom.

The CMR is situated in the Carajás Mineral Province (Figure 1a,b), i.e., a major Neoarchean tectonic province of the Amazonian Craton, where banded iron formations and metavolcano–sedimentary sequences represent the middle-upper geological succession of this province [33]. The iron-rich deposits were formed during the successive weathering events of these rocks in the Cretaceous–Paleogene transition, which occurred under humid paleoclimate conditions, allowing the formation of extensive mature lateritic profiles [34,35].

These crusts contain a variety of geoenvironments with unique geological, geomorphological, and pedological characteristics, which influence the nutrient availability and energy flows within an essentially open system [12], as follows (Figure 1b,d): (1) slopes with rupestrian *canga* vegetation over plinthosols; (2) forested slopes and caves over plinthosols and ferralsols; (3) poorly drained depressions and levels covered by grasslands over plinthosols and histosols; as well as (4) doliniform lakes with organic mud sediment at the bottom.

The climate is characterized by two distinct seasons: a rainy season and a dry season [38]. During the rainy season, which lasts from November to May, the total rainfall ranges from 1545 mm to 1863 mm. In contrast, the dry season, occurring from June to October, receives between 159 mm and 321 mm of rainfall. The average recorded temperature is 27.2 °C, with a minimum of 26.6 °C in January and a maximum of 28.1 °C in September [39].

## 2. Materials and Methods

The examined specimens are stored in the herbaria of the Museu Paraense Emílio Goeldi (MPEG), Bioparque Vale Amazônia (HCJS), and Instituto Nacional de Pesquisas da Amazônia (INPA). Mature flower buds were extracted from the exsiccate collections and treated using standard pollen preparation methods. This included extracting flower buds from duplicate specimens before anthesis, followed by fixation in acetic acid and acetolysis [40]. For light microscopy, the pollen was mounted in glycerol jelly and sealed with paraffin [40,41]. All prepared slides were deposited in the Palinoteca of the Instituto Tecnológico Vale (PALIITV). Subsequently, the grains were examined, measured, and photographed using a Zeiss AXIO Imager M2 microscope with a Pan-APOCHROMAT 20×, 40×, and 100× objective (Carl Zeiss Microscopy GmbH, Oberkochen, Germany). The descriptive palynology terminology used in this study is as follows [42]. Main morphological parameters are abbreviated as follows: polar diameter (P); equatorial diameter (E); or simply diameter (D). These variables were examined in 20 grains per sample [21].

The types are grouped into ferns and lycophytes, monocots, eudicots, and magnoliids. Within each group, plant families are alphabetically organized according to [43,44], as well as their corresponding species. Species names, life forms, and habitats were verified by consulting the Brazilian Species Database of the Flora of the Carajás project [16] and REFLORA [45]. The geoenvironments terminology of [12] was applied according to the habitats and geographic coordinates of each species available in the cited database. Pollination syndromes according to [46], the degree of domestication [47], and main uses [48] were also included (Table S1). It is important to note that the ecological discussion of geoenvironments and pollination syndromes in this study only pertains to a selection of plant species. Specifically, it focuses on the 204 plant species included in the palynological atlas of Amazon *canga* vegetation and does not encompass the entire floristic diversity of the study area.

## 3. Results

The ferns and lycophytes present 10 species, 8 genera, and 5 families of the Polypodiales Link, Hort. BTerol., and 2 species of Isoëtales Prantl, Lehrb. Monocots are represented by 6 orders, encompassing 12 families, 36 genera, and 63 species. The magnoliids order is only represented by 2 species, and the genera of the Annonaceae Juss. Eudicots are the most representative of this pollen atlas, with 130 species, 91 genera, and 38 families. Some descriptive parameters are summarized in Table 1. Table S1 provides a summary of the plant species from the *canga* vegetation whose pollen and spore grains are described in this atlas. A dichotomous key can be found in the Supplementary Materials (Data S1).

### Description of Spores and Pollen

**Figure 2:**
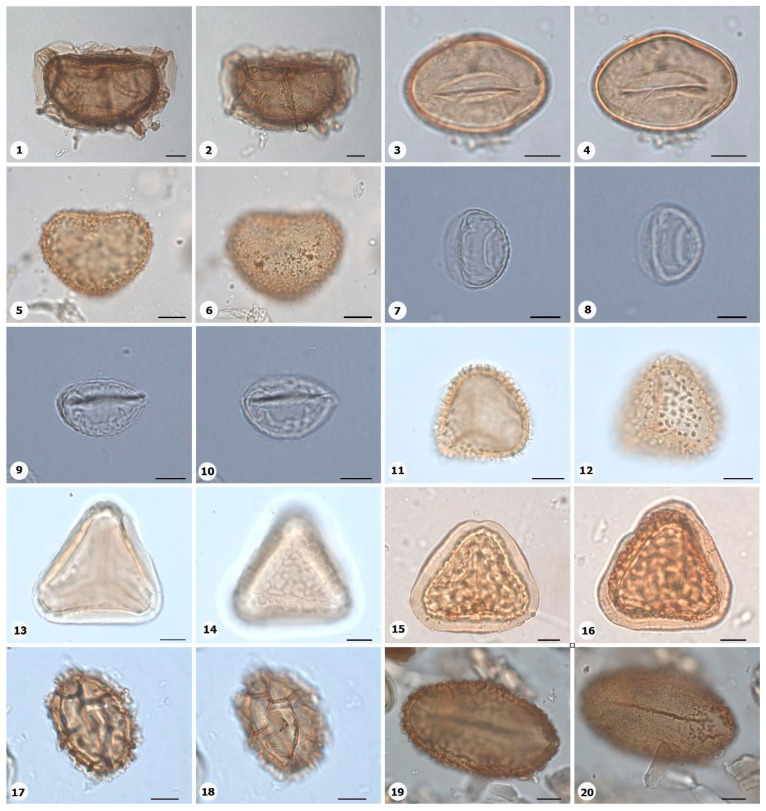
Ferns and lycophytes. 1–2: *Asplenium serratum*; 3–4: *Blechnum polypodioides*; 5–6: *Pteridium esculentum*; 7–8: *Isoëtes cangae*; 9–10: *Isoëtes serracarajasensis*; 11–12: *Hemionitis palmata*; 13–14: *Pteris denticulata*; 15–16: *Pteris pungens*; 17–18: *Christella hispidula*; 19–20: *Cyclosorus interruptus*. Scale: 10 µm.

**FERNS AND LYCOPHYTES**
***Order*: Polypodiales Link**
***Family*: Aspleniaceae Newman**
***Species*: * Asplenium serratum* L. (Figure 2: 1–2)**
  Herbarium number: HCJS 1548
  Description: Spores single, shape with rounded ends; laesura straight, margo narrow; sclerine ~3 µm thick, exospore distinct, ornamentation with thin and irregular undulating membrane, resembling a hamulate pattern.
Life form: Herb.
Geoenvironment: Forested slopes and caves over plinthosols and ferralsols.

***Order*: Polypodiales Link**
***Family*: Blechnaceae Newman**
***Species*: * Blechnum polypodioides* Raddi (Figure 2: 3–4)**
  Herbarium number: HCJS 4365
  Description: Spores single, shape with rounded ends; laesura straight, margo 2–3 µm thick, protruding.
  Life form: Herb.
  Geoenvironment: Forested slopes and caves over plinthosols and ferralsols.

***Family*: Dennstaedtiaceae Lotsy**
***Species*: * Pteridium esculentum* (G. Forst.) Cockayne (Figure 2: 5–6)**
  Herbarium number: BHCB 601
  Description: Spores single, shape with rounded ends; laesura indistinct; exospore 1.5–2.4 thick.
  Life form: Herb.
  Geoenvironment: Upland anthropic areas.

***Order*: *Isoëtales* C.Agardh**
***Family*: *Isoëtaceae* Rchb.f.**
***Species*: * Isoëtes cangae* J.B.S.Pereira, Salino and Stützel (Figure 2: 7–8)**
  Herbarium number: HCJS 6277
  Description: Spores single, shape with pointed ends; laesura straight, without prominent invagination; perispore microechinate in proximal view and microechinate to tuberculate in distal view.
  Life form: Herb.
  Geoenvironment: Doliniform lakes; Amendoim Lake, S11D, active lake
***Species*: * Isoëtes serracarajensis* J.B.S.Pereira, Salino and Stützel (Figure 2: 9–10)**
  Herbarium number: HCJS 5433
  Description: Spores single, shape with pointed ends; laesura straight, without prominent invagination; perispore laevigate in proximal view and microechinate to tuberculate in distal view.
  Life form: Herb
  Geoenvironment: Poorly drained depressions and levels over plinthosols and histosols.

***Order*: Polypodiales Link**
***Family*: Pteridaceae E.D.M.Kirchn.**
***Species*: * Hemionitis palmata* L. (Figure 2: 11–12)**
  Herbarium number: HCJS 1465
  Description: Spores single, shape with rounded corners; curvature absent, margo indistinct, commissure straight; endospore 1 µm thick; exospore 1 µm thick, echinae 1–2 µm height.
  Life form: Herb.
  Geoenvironment: Forested slopes and caves over plinthosols and ferralsols.
***Species*: * Pteris denticulata* Sw. (Figure 2: 13–14)**
  Herbarium number: HCJS 1007
  Description: Spores single, shape with rounded corners; curvature absent, commissure curved, margo ~3 µm thick, very distinct; endospore 1 µm thick, exospore 1 µm thick, cingulated, cingulum about 4–5 µm thick and likely varying in thickness until radial area.
  Life form: Herb.
  Geoenvironment: Forested slopes and caves over plinthosols and ferralsols.
***Species*: * Pteris pungens* Willd. (Figure 2: 15–16)**
  Herbarium number: HCJS 1559
  Description: Spores single, shape with rounded corners; margo indistinct, commissure straight; endospore indistinct, exospore 1 µm thick, cingulated, cingulum about 5 µm thick and not varying in thickness.
  Life form: Herb.
  Geoenvironment: Forested slopes and caves over plinthosols and ferralsols.

***Family*: Thelypteridaceae Pic.Serm.**
***Species*: * Christella hispidula* (Decne.) Holttum (Figure 2: 17–18)**
  Herbarium number: HCJS 4775
  Description: Spores single, shape with rounded ends; laesura straight to curved, margo indistinct; sclerine ~2–3 µm thick, exospore distinct.
  Life form: Herb.
  Geoenvironment: Forested slopes and caves over plinthosols and ferralsols.
***Species*: * Cyclosorus interruptus* (Willd.) H. Ito (Figure 2: 19–20)**
  Herbarium number: HCJS 3009
  Description: Spores single, shape with rounded ends; laesura straight to curved, margo indistinct; exospore 2 µm thick.
  Life form: Herb.
  Geoenvironment: Poorly drained depressions and levels over plinthosols and histosols.

**Figure 3:**
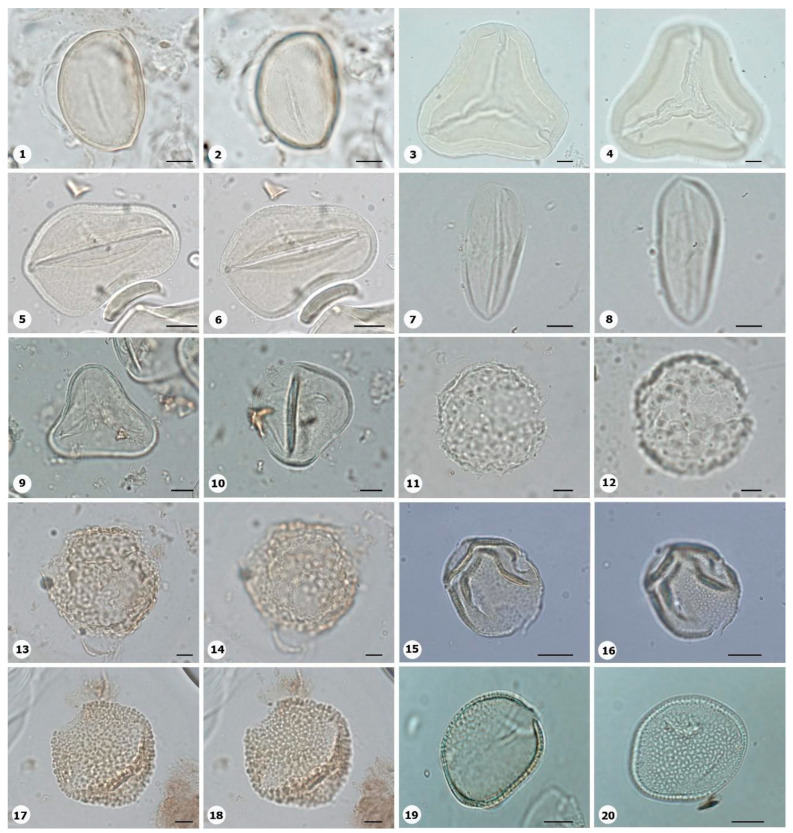
Monocots. 1–2: *Philodendron wullschlaegelii*; 3–4: *Acrocomia aculeata*; 5–6: *Attalea maripa*; 7–8: *Euterpe oleraceae*; 9–10: *Oenocarpus distichus*; 11–12: *Socratea exorrhiza*; 13–14: *Aechmea bromeliifolia*; 15–16: *Aechmea castelnavii*; 17–18: *Aechmea mertensii*; 19–20: *Dyckia duckei*. Scale: 10 µm.

**MONOCOTS**
***Order*: Alismatales R. Br. ex Bercht. and J. Presl**
***Family*: Araceae Juss.**
***Species*: * Philodendron wullschlaegelii* Schott (Figure 3: 1–2)**
  Herbarium number: MG 214008
  Description: Monads, large, sulci marginate; amb elliptical; exine 1.2 µm thick, tectate.
  Life form: Herb.
  Geoenvironment: Forested slopes and caves over plinthosols and ferralsols.

***Order*: Arecales Bromhead**
***Family*: Arecaceae Bercht. and J.Presl.**
***Species*: * Acrocomia aculeata* (Jacq.) Lodd. ex Mart. (Figure 3: 3–4)**
  Herbarium number: HCJS 0646
  Description: Monad, large; sulci marginate; amb triangular–obtuse–concave; exine 7.3 µm thick, tectate.
  Life form: Palm.

Geoenvironment: Forested slopes and caves over plinthosols and ferralsols.
***Species*: * Attalea maripa* (Aubl.) Mart. (Figure 3: 5–6)**
  Herbarium number: HCJS 0383
  Description: Monad, large; sulci marginate; amb elliptical; exine 2.6 µm thick, tectate.
  Life form: Palm.
  Geoenvironment: Forested slopes and caves over plinthosols and ferralsols.
***Species*: * Euterpe oleraceae* Mart. (Figure 3: 7–8)**
  Herbarium number: HCJS 0272
  Description: Monad, large; sulci with a slight margo; amb elliptical with acute ends; exine 1 µm thick, tecta indistinct.
  Life form: Palm.
  Geoenvironment: Poorly drained depressions and levels over plinthosols and histosols, doliniform lakes with organic mud sediment at the bottom.
***Species*: * Oenocarpus distichus* Mart. (Figure 3: 9–10)**
  Herbarium number: HCJS 0370
  Description: Monad, medium; sulci with a slight margo; amb triangular–obtuse–concave; exine 2–2.3 µm thick, tectate.
  Life form: Palm.
  Geoenvironment: Forested slopes and caves over plinthosols and ferralsols.
***Species*: * Socratea exorrhiza* (Mart.) H.Wendl. (Figure 3: 11–12)**
  Herbarium number: HCJS 1433
  Description: Monad, apolar; circular; exine 3.5–5 µm thick, tectate.
  Life form: Palm.
  Geoenvironment: Forested slopes and caves over plinthosols and ferralsols.

***Order*: Poales Small**
***Family*: Bromeliaceae Juss.**
***Species*: *Aechmea bromeliifolia* (Rudge) Baker (Figure 3: 13–14)**
  Herbarium number: HCJS 1335
  Description: Monad, large; large pores 7–8 µm diameter; circular; exine 2.5 µm thick.
  Life form: Herb.
  Geoenvironment: Forested slopes and caves over plinthosols and ferralsols, slopes with *canga* vegetation over plinthosols.
***Species*: * Aechmea castelnavii* Baker (Figure 3: 15–16)**
  Herbarium number: MG 214066
  Description: Monad, medium; spheroidal; tectate, columellate, exine 1.2 µm thick, heterobrochate reticulate.
  Life form: Herb.
  Geoenvironment: Forested slopes and caves over plinthosols and ferralsols, slopes with *canga* vegetation over plinthosols.
***Species*: * Aechmea mertensii* (G.Mey.) Schult. and Schult.f. (Figure 3: 17–18)**
  Herbarium number: HCJS 1410
  Description: Monad, large; exine 4.2 µm thick, tectate, heterobrochate reticulate.
  Life form: Herb.
  Geoenvironment: Forested slopes and caves over plinthosols and ferralsols.
***Species*: * Dyckia duckei* L.B.Sm. (Figure 3: 19–20)**
  Herbarium number: MG 222350; HCJS 1040
  Description: Monad, medium; margo indistinct; amb elliptical; exine 1.6 µm thick, tectate, heterobrochate reticulate.
  Life form: Herb.
  Geoenvironment: Slopes with *canga* vegetation over plinthosols.

**Figure 4:**
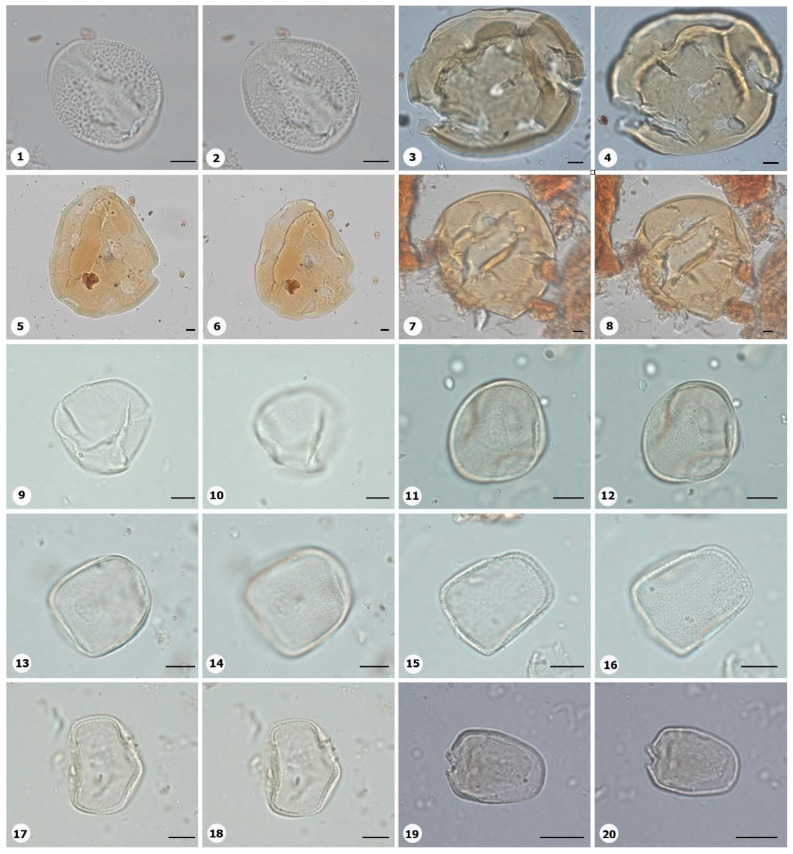
Monocots. 1–2: *Pitcarnia lanuginosa*. 3–4: *Chamaecostus acualis*; 5–6: *Chamaecostus lanceolatus*; 7–8: *Costus scaber*; 9–10: *Bulbostylis paraensis*; 11–12: *Cyperus aggregatus*; 13–14: *Cyperus amabilis*; 15–16: *Cyperus haspan*; 17–18: *Cyperus laxus*; 19–20: *Cyperus sphacelatus*. Scale: 10 µm.

***Species*: * Pitcairnia lanuginosa* Ruiz & Pav. (** Figure 4 **: 1–2)**
  Herbarium number: MG 222323
  Description: Monad, medium; margo indistinct; amb elliptical; exine 1 µm thick, tectate, heterobrochate reticulate.
  Life form: Herb.
  Geoenvironment: Slopes with *canga* vegetation over plinthosols.

***Order*: Zingiberales Grisebach**
***Family*: Costaceae Nakai**
***Species*: * Chamaecostus acaulis* (S.Moore) T.André & C.D.Specht (Figure 4: 3–4)**
  Herbarium number: INPA 257238, 109585
  Description: Monad, very large; exine 4–5 µm thick, tectate.
  Life form: Herb.
  Geoenvironment: Forested slopes and caves over plinthosols and ferralsols, slopes with *canga* vegetation over plinthosols.
***Species*: * Chamaecostus lanceolatus* (Petersen) C.D.Specht and D.W.Stey (Figure 4: 5–6)**
  Herbarium number: INPA 98074, 53463
  Description: Monad, very large; pores slightly annulate; spheroidal; exine 5–6 µm thick, tectate.
  Life form: Herb.
  Geoenvironment: Forested slopes and caves over plinthosols and ferralsols, slopes with *canga* vegetation over plinthosols.
***Species*: * Costus scaber* Ruiz & Pav. (Figure 4: 7–8)**
  Herbarium number: INPA 163081, 262903
  Description: Monad, gigantic; exine 5 µm thick, tectate.
  Life form: Herb.
  Geoenvironment: Forested slopes and caves over plinthosols and ferralsols, slopes with *canga* vegetation over plinthosols.

***Order*: Poales Small**
***Family*: Cyperaceae Juss.**
***Species*: * Bulbostylis paraensis* C.B.Clarke (Figure 4: 9–10)**
  Herbarium number: BHCB 115277
  Description: Monad, medium; exine 1 µm thick, tectate.
  Life form: Herb.
  Geoenvironment: Forested slopes and caves over plinthosols and ferralsols, poorly drained depressions and levels over plinthosols and histosols.
***Species*: * Cyperus aggregatus* (Willd.) Endl. (Figure 4: 11–12)**
  Herbarium number: BHCB 137680
  Description: Monad, medium; presence of pseudoapertures; exine 1.5 µm thick, tectate.
  Life form: Herb.
  Geoenvironment: Slopes with *canga* vegetation over plinthosols, forested slopes and caves over plinthosols and ferralsols, poorly drained depressions and levels over plinthosols and histosols.
***Species*: * Cyperus amabilis* Vahl (Figure 4: 13–14)**
  Herbarium number: BHCB 115278
  Description: Monad, medium; presence of pseudoapertures; exine 1.5 µm thick, tectate.
  Life form: Herb.
  Geoenvironment: Slopes with *canga* vegetation over plinthosols, forested slopes and caves over plinthosols and ferralsols, poorly drained depressions and levels over plinthosols and histosols.
***Species*: * Cyperus haspan* L. (Figure 4: 15–16)**
  Herbarium number: BHCB 139491, MG 214017
  Description: Monad, medium; presence of pseudoapertures; proximal pole slightly smaller than distal pole; exine 1.3 µm thick, tectate.
  Life form: Herb.
  Geoenvironment: Poorly drained depressions and levels over plinthosols and histosols, forested slopes and caves over plinthosols and ferralsols, slopes with *canga* vegetation over plinthosols, doliniform lakes with organic mud sediments at the bottom.
***Species*: * Cyperus laxus* Lam. (Figure 4: 17–18)**
  Herbarium number: BHCB 137669
  Description: Monad, medium; proximal pole slightly smaller than distal pole; exine 1.3 µm thick, tectate.
  Life form: Herb.
  Geoenvironment: Forested slopes and caves over plinthosols and ferralsols, slopes with *canga* vegetation over plinthosols.
***Species*: * Cyperus sphacelatus* Rottb. (Figure 4: 19–20)**
  Herbarium number: MG 214013
  Description: Monad, small; proximal pole slightly smaller than distal pole; exine 0.5–0.6 µm thick, tectate.
  Life form: Herb.
  Geoenvironment: Slopes with *canga* vegetation over plinthosols.

**Figure 5:**
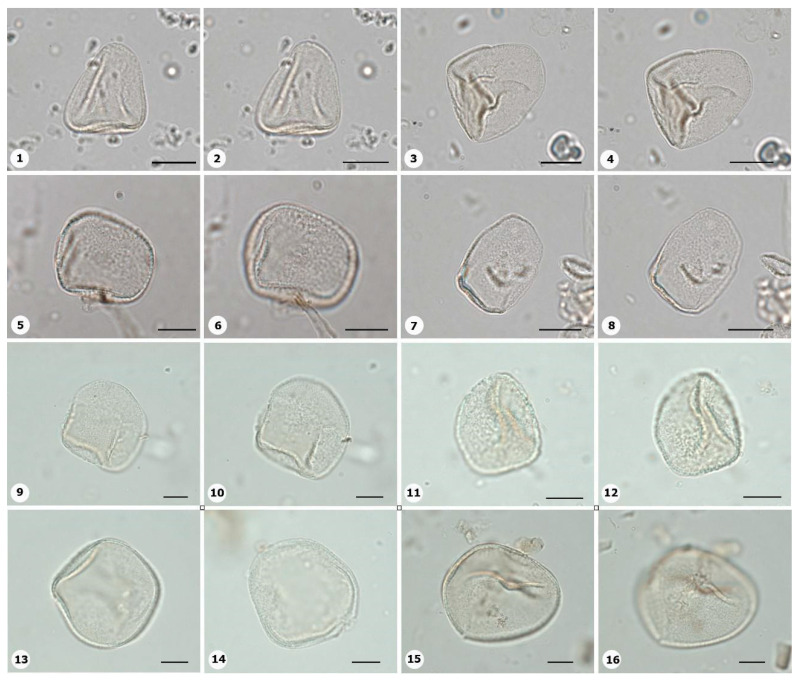
Monocots. 1–2: *Cyperus surinamensis*; 3–4: *Eleocharis flavescens*; 5–6: *Rhynchospora barbata*; 7–8: *Rhynchospora corymbosa*; 9–10: *Rhynchospora seccoi*; 11–12: *Rhynchospora tenuis*; 13–14: *Scleria cyperina*; 15–16: *Scleria verticillata*.

***Species*: * Cyperus surinamensis* Rottb. (Figure 5: 1–2)**
  Herbarium number: BHCB 142679
  Description: Monad, small; proximal pole smaller than distal pole; exine 0.8–0.9 µm thick, tectate.
  Life form: Herb.
  Geoenvironment: Slopes with *canga* vegetation over plinthosols.
***Species*: * Eleocharis flavescens* (Poir.) Urb. (Figure 5: 3–4)**
  Herbarium number: MG 214003
  Description: Monad, medium, heteropolar; inaperturate; elliptical, proximal pole smaller than distal pole; exine 0.8 µm thick, tectate, ornamentation microreticulate.
  Life form: Herb.
  Geoenvironment: Slopes with *canga* vegetation over plinthosols.
***Species*: * Rhynchospora barbata* (Vahl) Kunth (Figure 5: 5–6)**
  Herbarium number: BHCB 130685, MG 213983, 222341
  Description: Monad, medium; proximal pole slightly smaller than distal pole; exine 1.5 µm thick, tectate, ornamentation with coarse muri.
  Life form: Herb.
  Geoenvironment: Slopes with *canga* vegetation over plinthosols, forested slopes and caves over plinthosols and ferralsols, poorly drained depressions and levels over plinthosols and histosols.
***Species*: * Rhynchospora corymbosa* (L.) Britton (Figure 5: 7–8)**
  Herbarium number: BHCB 115300
  Description: Monad, medium; proximal pole slightly smaller than distal pole; exine 1 µm thick, tectate.
  Life form: Herb.
  Geoenvironment: Forested slopes and caves over plinthosols and ferralsols, poorly drained depressions and levels over plinthosols and histosols.
***Species*: * Rhynchospora seccoi* C.S. Nunes, P.J.S. Silva Filho & A. Gil (Figure 5: 9–10)**
  Herbarium number: HCJS 0732, HCJS 0791
  Description: Monad, medium; exine 1–1.2 µm thick, tectate.
  Life form: Herb.
  Geoenvironment: Slopes with *canga* vegetation over plinthosols, poorly drained depressions and levels over plinthosols and histosols.

***Species*: * Paepalanthus aff*. *fasciculatus* (Figure 5: 11–12)**
  Herbarium number: BHCB 137678, BHCB 155861
  Description: Monad, medium; exine 1 µm thick, tectate.
  Life form: Herb.
  Geoenvironment: Forested slopes and caves over plinthosols and ferralsols, poorly drained depressions and levels over plinthosols and histosols.
***Species*: * Scleria cyperina* Willd. ex Kunth (Figure 5: 13–14)**
  Herbarium number: MG 214081
  Description: Monad, medium; exine 1–1.2 µm thick, tectate.
  Life form: Herb.
  Geoenvironment: Slopes with *canga* vegetation over plinthosols, poorly drained depressions and levels over plinthosols and histosols.
***Species*: * Scleria verticillata* Muhl. ex Willd. (Figure 5: 15–16)**
  Herbarium number: BHCB 158212
  Description: Monad, medium; exine 1 µm thick, tectate.
  Life form: Herb.
  Geoenvironment: Slopes with *canga* vegetation over plinthosols, poorly drained depressions and levels over plinthosols and histosols.

**Figure 6:**
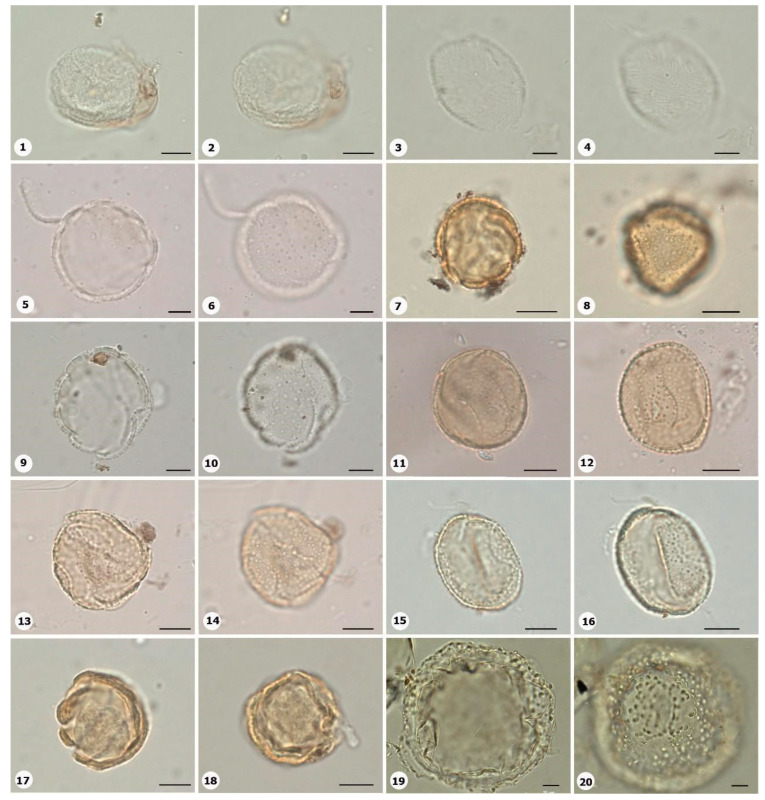
Monocots. 1–2: *Dioscorea glandulosa*. 3–4: *Dioscorea pohlii*; 5–6: *Eriocaulon aff. setaceum*; 7–8 *Eriocaulon setaceum*; 9–10*: Paepalanthus aff. fasciculatus*; 11–12: *Syngonanthus caulescens*; 13–14: *Syngonanthus discretifolius*; 15–16: *Syngonanthus heteropeplus*; 17–18: *Syngonanthus* sp. 1; 19–20: *Heliconia adeliana*. Scale: 10 µm.

***Order*: *Dioscoreales* R.Br.**
**Family: Dioscoreaceae (R. Br.)**
***Species*: * Dioscorea glandulosa* (Klotzsch ex Griseb.) Kunth (Figure 6: 1–2)**
  Herbarium number: HCJS 0806
  Description: Monad, medium; exine 0.6 µm thick, tectate.
  Life form: Liana.
  Geoenvironment: Forested slopes and caves over plinthosols and ferralsols, slopes with *canga* vegetation over plinthosols.
***Species*: * Dioscorea pohlii* Griseb. (Figure 6: 3–4)**
  Herbarium number: HCJS 0482
  Description: Monad, large; exine 1.8–2 µm thick, tectate.
  Life form: Liana.
  Geoenvironment: Slopes with *canga* vegetation over plinthosols.

***Order*: Poales Small**
***Family*: Eriocaulaceae Martinov**
***Species*: * Eriocaulon aff. setaceum* L. (Figure 6: 5–6)**
  Herbarium number: HCJS 1578
  Description: Monad, medium; spiral apertures in a crosshatch pattern, with 2–3 apertures; exine 1.8–2 µm thick, tectate.
  Life form: Herb.
  Geoenvironment: Poorly drained depressions and levels over plinthosols and histosols.
***Species*: * Eriocaulon setaceum* L. (Figure 6: 7–8)**
  Herbarium number: MG 214910
  Description: Monad, small; spiral apertures in a crosshatch pattern, with 2–3 apertures; exine 1.2–1.3 µm thick, tectate.
  Life form: Herb.
  Geoenvironment: Poorly drained depressions and levels over plinthosols and histosols.
***Species*: * Paepalanthus aff*. *fasciculatus* (Rottb.) Kunth (Figure 6: 9–10)**
  Herbarium number: MG 85842
  Description: Monad, small; spiral apertures in a crosshatch pattern, with 2–3 apertures; exine 1.7–1.8 µm thick, tectate.
  Life form: Herb.
  Geoenvironment: Poorly drained depressions and levels over plinthosols and histosols.
***Species*: * Syngonanthus caulescens* (Poir.) Ruhland (Figure 6: 11–12)**
  Herbarium number: HCJS 0541, MG 223908
  Description: Monad, small; spiral apertures with apertures tracing various designs (many arrangements); spheroidal; exine 1 µm thick, tectate.
  Life form: Herb.
  Geoenvironment: Forested slopes and caves over plinthosols and ferralsols, poorly drained depressions and levels over plinthosols and histosols.
***Species*: * Syngonanthus discretifolius* (Moldenke) M.T.C.Watan. (Figure 6: 13–14)**
  Herbarium number: MG 214016
  Description: Monad, small; spiral apertures with apertures tracing various designs (many arrangements); exine 1 µm thick, tectate.
  Life form: Herb.
  Geoenvironment: Poorly drained depressions and levels over plinthosols and histosols.
***Species*: * Syngonanthus heteropeplus* (Koern.) Ruhland (Figure 6: 15–16)**
  Herbarium number: MG 117013
  Description: Monad, small; spiral apertures with apertures tracing various designs (many arrangements); exine 1 µm thick, tectate.
  Life form: Herb
  Geoenvironment: Poorly drained depressions and levels over plinthosols and histosols.
***Species*: * Syngonanthus* sp. 1 (Figure 6: 17–18)**
  Collection number: ITV 1774
  Description: Monad, small; spiral apertures with apertures tracing various designs (many arrangements); exine 1.2–1.3 µm thick, tectate.
  Life form: Herb.
  Geoenvironment: Forested slopes and caves over plinthosols and ferralsols, poorly drained depressions and levels over plinthosols and histosols.

***Order*: Zingiberales Grisebach**
***Family*: Heliconiaceae Nakai**
***Species*: * Heliconia adeliana* Emygdio & E.Santos (Figure 6: 19–20)**
  Herbarium number: MG 251426
  Description: Monad, large; exine 6.5–7 µm thick, nexine significantly thicker than endexine, tectate.
  Life form: Herb.
  Geoenvironment: Forested slopes and caves over plinthosols and ferralsols.

**Figure 7:**
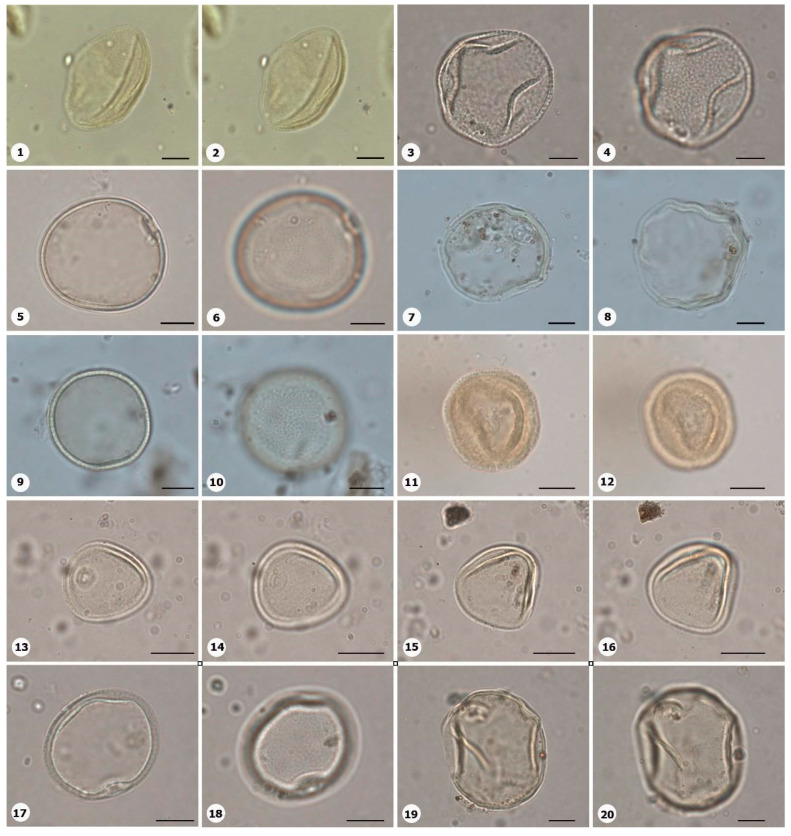
Monocots. 1–2: *Mayaca fluviatilis*; 3–4: *Axonopus capillaris*; 5–6: *Axonopus carajasensis*; 7–8: *Axonopus longispicus*; 9–10 *Eragrostis maypurensis*; 11–12: *Eragrostis rufescens*; 13–14: *Hildaea breviscrobs*; 15–16: *Ichnanthus calvescens*; 17–18: *Isachne polygonoides*; 19–20: *Mesosetum cayennense*. Scale: 10 µm.

***Order*: Poales Small**
***Family*: Mayacaceae *Kunth***
***Species*: * Mayaca fluviatilis* Aubl. (Figure 7: 1–2)**
  Herbarium number: MG 222303
  Description: Monad, medium; exine 1.5–1.6 µm thick, tectate.
  Life form: Herb.
  Geoenvironment: Poorly drained depressions and levels over plinthosols and histosols, doliniform lakes with organic mud sediment at the bottom.
***Family*: Poaceae Barnhart**
***Species*: * Axonopus capillaris* (Lam.) Chase (Figure 7: 3–4)**
  Herbarium number: MG 116782
  Description: Monad, medium; pores annulate; exine 1.1–2 µm thick, tectate.
  Life form: Herb.
  Geoenvironment: Forested slopes and caves over plinthosols and ferralsols, slopes with *canga* vegetation over plinthosols.
***Species*: * Axonopus carajasensis* Bastos (Figure 7: 5–6)**
  Herbarium number: BHCB 155944
  Description: Monad, medium; pores annulate; exine 0.7–0.8 µm thick, tectate.
  Life form: Herb.
  Geoenvironment: Slopes with *canga* vegetation over plinthosols.
***Species*: * Axonopus longispicus* (Döll) Kuhlm (Figure 7: 7–8)**
  Herbarium number: MG 99379
  Description: Monad, medium; pores annulate; exine 0.9–1.5 µm thick, tectate.
  Life form: Herb.
  Geoenvironment: Slopes with *canga* vegetation over plinthosols.
***Species*: * Eragrostis maypurensis* (Kunth) Steud (Figure 7: 9–10)**
  Herbarium number: MG 147039
  Description: Monad, medium; pores annulate; exine 0.8–1.3 µm thick, tectate.
  Life form: Herb.
  Geoenvironment: Slopes with *canga* vegetation over plinthosols.

***Species*: * Eragrostis rufescens* Schrad. ex Schult. (Figure 7: 11–12)**
  Herbarium number: MG 213985
  Description: Monad, medium; pores annulate; exine 1.5 µm thick, tectate.
  Life form: Herb.
  Geoenvironment: Slopes with *canga* vegetation over plinthosols.
***Species*: * Hildaea breviscrobs* (Döll) C.Silva & R.P. Oliveira (Figure 7: 13–14)**
  Herbarium number: HCJS 1740
  Description: Monad, medium; pores annulate; exine 1.3–1.5 µm thick, tectate.
  Life form: Herb.
  Geoenvironment: Forested slopes and caves over plinthosols and ferralsols.
***Species*: * Ichnanthus calvescens* (Nees ex Trin.) Döll (Figure 7: 15–16)**
  Herbarium number: MG 214084
  Description: Monad, medium; pores annulate; exine 1.3–1.5 µm thick, tectate.
  Life form: Herb.
  Geoenvironment: Forested slopes and caves over plinthosols and ferralsols, slopes with *canga* vegetation over plinthosols.
***Species*: * Isachne polygonoides* (Lam.) Döll (Figure 7: 17–18)**
  Herbarium number: MG 222316
  Description: Monad, medium; pores annulate; exine 1 µm thick, tectate.
  Life form: Herb.
  Geoenvironment: Slopes with *canga* vegetation over plinthosols, poorly drained depressions and levels over plinthosols and histosols.
***Species*: * Mesosetum cayennense* Steud. (Figure 7: 19–20)**
  Herbarium number: MG 105630
  Description: Monad, medium; pores annulate; exine 0.9–1.4 µm thick, tectate.
  Life form: Herb.
  Geoenvironment: Slopes with *canga* vegetation over plinthosols, forested slopes and caves over plinthosols and ferralsols, poorly drained depressions and levels over plinthosols and histosols.

**Figure 8:**
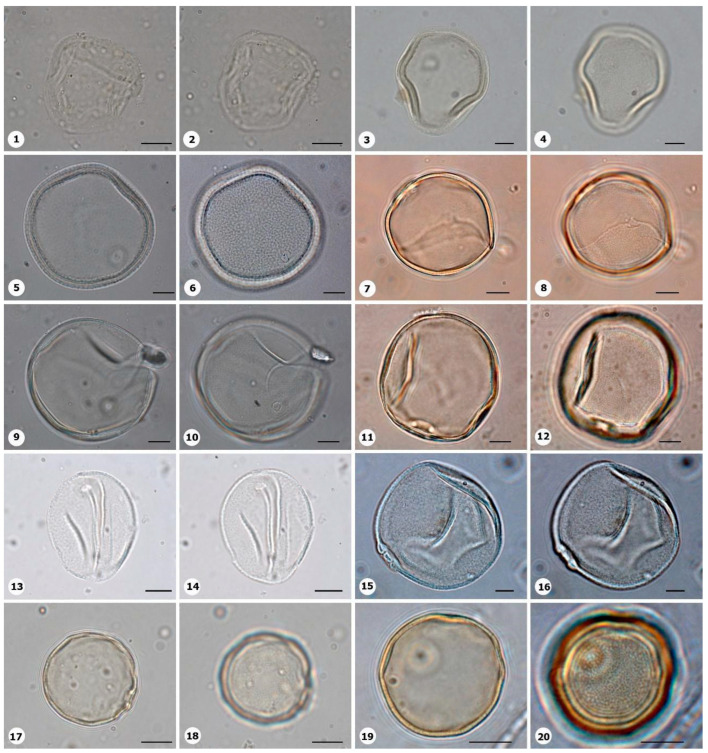
Monocots. 1–2: *Mnesithea aurita*. 3–4: *Otachyrium versicolor*; 5–6: *Paspalum carajasense*; 7–8: *Paspalum carinatum*; 9–10 *Paspalum virgatum*; 11–12: *Rhytachne gonzalezii*; 13–14: *Sporobolus multiramosus*; 15–16: *Trichanthecium parvifolium*; 17–18: *Trichanthecium polycomum*; 19–20: *Trichanthecium* sp. 1. Scale: 10 µm.

***Species*: * Mnesithea aurita* (Steud.) de Koning & Sosef (Figure 8: 1–2)**
  Herbarium number: MG 67183
  Description: Monad, medium; pores annulate; exine 0.8–1 µm thick, tectate.
  Life form: Herb.
  Geoenvironment: Slopes with *canga* vegetation over plinthosols.
***Species*: * Otachyrium versicolor* (Döll) Henrard (Figure 8: 3–4)**
  Herbarium number: BHCB 139242
  Description: Monad, medium; pores annulate; exine 1–1.4 µm thick, tectate.
  Life form: Herb.
  Geoenvironment: Poorly drained depressions and levels over plinthosols and histosols.
***Species*: * Paspalum carajasense* S.Denham (Figure 8: 5–6)**
  Herbarium number: MG 208828
  Description: Monad, large; pores annulate but some grains are indistinct; exine 2–2.8 µm thick, tectate.
  Life form: Herb.
  Geoenvironment: Slopes with *canga* vegetation over plinthosols.
***Species*: * Paspalum carinatum* Humb. & Bonpl. ex Flüggé (Figure 8: 7–8)**
  Herbarium number: MG 214010
  Description: Monad, medium; pores annulate; exine 1.5–3 µm thick, tectate.
  Life form: Herb.
  Geoenvironment: Slopes with *canga* vegetation over plinthosols.
***Species*: * Paspalum virgatum* L. (Figure 8: 9–10)**
  Herbarium number: MG 67531
  Description: Monad, medium; pores annulate; exine 0.9–1.7–3 µm thick, tectate.
  Life form: Herb.
  Geoenvironment: Poorly drained depressions and levels over plinthosols and histosols.

***Species*: * Rhytachne gonzalezii* Davidse (Figure 8: 11–12)**
  Herbarium number: MG 135244
  Description: Monad, medium; pores annulate; exine 1.3–3.4 µm thick, tectate.
  Life form: Herb.
  Geoenvironment: Poorly drained depressions and levels over plinthosols and histosols.
***Species*: * Sporobolus multiramosus* Longhi-Wagner & Boechat (Figure 8: 13–14)**
  Herbarium number: MG 213981
  Description: Monad, medium; pores annulate; exine 1.1–1.8 µm thick, tectate.
  Life form: Herb.
  Geoenvironment: Forested slopes and caves over plinthosols and ferralsols.
***Species*: * Trichanthecium parvifolium* (Lam.) Zuloaga & Morrone (Figure 8: 15–16)**
  Herbarium number: MG 213975
  Description: Monad, medium; pores with operculum, annulate; exine 1.1–1.7 µm thick, tectate.
  Life form: Herb.
  Geoenvironment: Slopes with *canga* vegetation over plinthosols, poorly drained depressions and levels over plinthosols and histosols.
***Species*: * Trichanthecium polycomum* (Trin.) Zuloaga & Morrone (Figure 8: 17–18)**
  Herbarium number: MG 215537
  Description: Monad, medium; pores annulate; exine 1–1.5 µm thick, tectate.
  Life form: Herb.
  Geoenvironment: Poorly drained depressions and levels over plinthosols and histosols.
***Species*: * Trichanthecium* sp. 1 (Figure 8: 19–20)**
  Herbarium number: MG 213989
  Description: Monad, medium; pores annulate; exine 1–1.5 µm thick, tectate.
  Life form: Herb.
  Geoenvironment: Slopes with *canga* vegetation over plinthosols, poorly drained depressions and levels over plinthosols and histosols.

**Figure 9:**
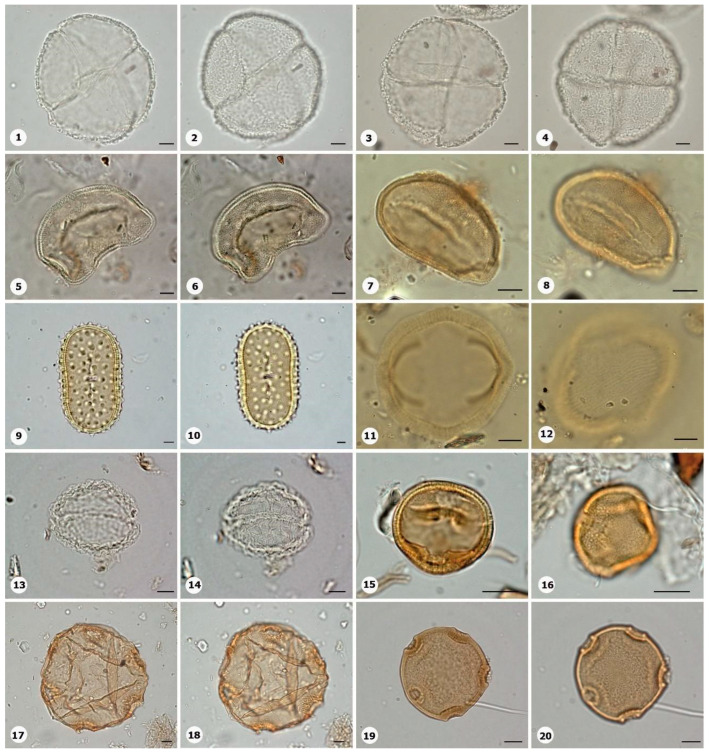
Monocots, Eudicots, and Magnoliids. 1–2: *Vellozia* sp. 1; 3–4: *Vellozia* sp. 2; 5–6: *Xyris brachysepala*; 7–8: *Xyris macrocephala*; 9–10: *Justicia birae*; 11–12: *Anacardium occidentale*; 13–14: *Onychopetalum amazonicum*; 15–16: *Xylopia aromática*; 17–18: *Mandevilla hirsuta*; 19–20: *Mandevilla tenuifolia*. Scale: 10 µm.

***Order*: Liliales Perleb**
***Family*: Velloziaceae *J.Agardh***
***Species*: * Vellozia* sp. 1 (Figure 9: 1–2)**
  Collection code: ITV 2114
  Description: Tetragonal tetrad, large; exine 2.5–3 µm.
  Life form: Shrub and herb.
  Geoenvironment: Slopes with *canga* vegetation over plinthosols.
***Species*: * Vellozia* sp. 2 (Figure 9: 3–4)**
  Collection code: ITV 2115
  Description: Tetragonal tetrad, large; exine 2–2.5 µm, reticulate with lumina of 4–5 µm width.
  Life form: Shrub and herb.
  Geoenvironment: Slopes with *canga* vegetation over plinthosols.

***Order*: Poales Small**
***Family*: Xyridaceae *C.Agardh***
***Species*: * Xyris brachysepala* Kral. (Figure 9: 5–6)**
  Herbarium number: MG 213994
  Description: Monads, large; exine 3 µm thick, tectate.
  Life form: Herb.
  Geoenvironment: Poorly drained depressions and levels over plinthosols and histosols, doliniform lakes with organic mud sediments at the bottom.
***Species*: * Xyris macrocephala* Vahl (Figure 9: 7–8)**
  Herbarium number: MG 214073
  Description: Monads, medium; exine 1–2 µm thick, tectate.
  Life form: Herb.
  Geoenvironment: Poorly drained depressions and levels over plinthosols and histosols, doliniform lakes with organic mud sediments at the bottom.

**EUDICOTS AND MAGNOLIIDS**
***Order*: Lamiales Bromhead**
***Family*: Acanthaceae Juss.**
***Species*: * Justicia birae* A.S.Reis, F.A.Silva, A.Gil & Kameyama (Figure 9: 9–10)**
  Herbarium number: HCJS 0728
  Description: Monads, large; brevicolpi; amb elliptical; exine 2–3 µm thick.
  Life form: Shrub and herb.
  Geoenvironment: Forested slopes and caves over plinthosols and ferralsols, slopes with *canga* vegetation over plinthosols.

***Order*: Sapindales Juss. ex Bercht. and J. Presl**
***Family*: Anacardiaceae R.Br.**
***Species*: * Anacardium occidentale* L. (Figure 9: 11–12)**
  Herbarium number: MG 112455
  Description: Monads, large; amb triangular–obtuse–convex; exine 5–5.6 µm thick, densely columellate, tectate.
  Life form: Tree.
  Geoenvironment: Forested slopes and caves over plinthosols and ferralsols, slopes with *canga* vegetation over plinthosols.

***Order*: Magnoliales Bromhead**
***Family*: Annonaceae Juss.**
***Species*: * Onychopetalum amazonicum* R.E.Fr. (Figure 9: 13–14)**
  Herbarium number: HCJS 1436
  Description: Monads, large; sulci with a prominent zonasulcus; amb elliptical; exine 1–1.5 µm thick.
  Life form: Tree.
  Geoenvironment: Forested slopes and caves over plinthosols and ferralsols, slopes with *canga* vegetation over plinthosols.
***Species*: * Xylopia aromatica* (Lam.) Mart. (Figure 9: 15–16)**
  Herbarium number: MG 59152
  Description: Monads, medium; pore costate, pore diameter 5 µm; amb circular; exine 2–3 µm thick.
  Life form: Tree and shrub.
  Geoenvironment: Forested slopes and caves over plinthosols and ferralsols, slopes with *canga* vegetation over plinthosols.

***Order*: Gentianales Juss. ex Bercht. & J.Presl**
***Family*: Apocynaceae Juss.**
***Species*: * Mandevilla hirsuta* (A.Rich.) K.Schum. (Figure 9: 17–18)**
  Herbarium number: MG 222310
  Description: Monads, very large; 4–5 pores with annuli; amb circular; exine 1.4–2 µm thick, ornamentation psilate and microreticulate near apertural region.
  Life form: Liana.
  Geoenvironment: Forested slopes and caves over plinthosols and ferralsols, slopes with *canga* vegetation over plinthosols.
***Species*: * Mandevilla tenuifolia* (J.C.Mikan) Woodson (Figure 9: 19–20)**
  Herbarium number: MG 57360
  Description: Monads, medium; five pores with prominent annuli; amb circular; exine 2–3 µm thick, tectate.
  Life form: Liana.
  Geoenvironment: Poorly drained depressions and levels over plinthosols and histosols.

**Figure 10:**
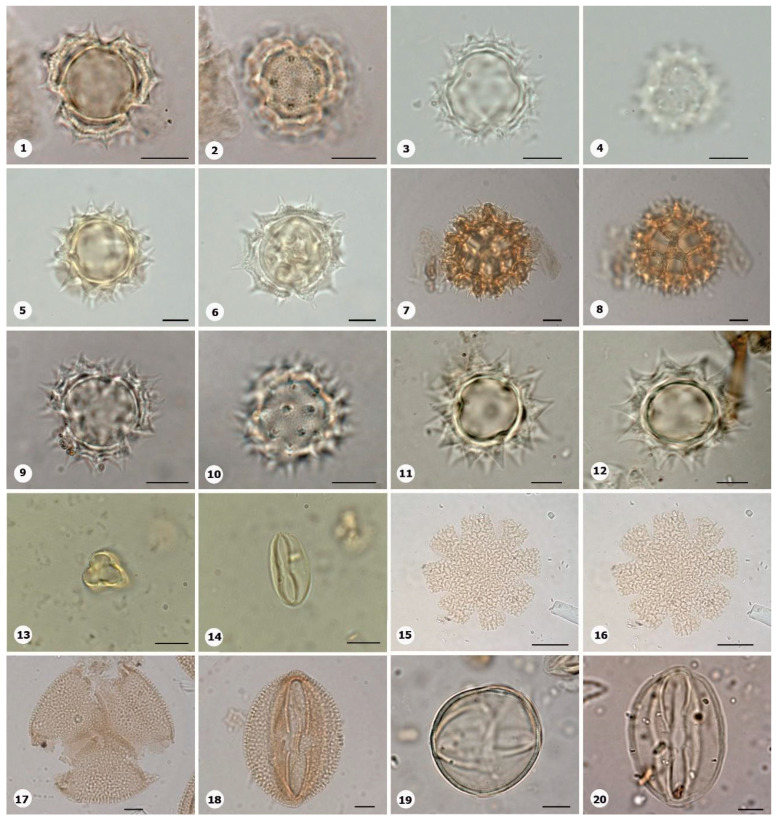
Eudicots. 1–2: *Cavalcantia glomerata*; 3–4: *Emilia* sp. 1; 5–6: *Ichthyothere terminalis*; 7–8: *Lepidaploa paraensis*; 9–10: *Monogereion carajensis*; 11–12: *Riencourtia pedunculosa*; 13–14: *Begonia guaduensis*; 15–16: *Anemopaegma carajasense*; 17–18: *Handroanthus serratifolius*; 19–20: *Jacaranda* copaia. Scale: 10 µm.

***Order*: Asterales Link**
***Family*: Asteraceae Bercht. and J.Presl**
***Species*: * Cavalcantia glomerata* (G.M.Barroso and R.M.King) R.M.King and H.Rob. (Figure 10: 1–2)**
  Herbarium number: MG 37869
  Description: Monad, small; brevicolpi; amb circular; exine 3–4 µm thick, densely columellate.
  Life form: Herb.
  Geoenvironment: Forested slopes and caves over plinthosols and ferralsols.
***Species*: * Emilia* sp. 1 (Cass.) Cass. (Figure 10: 3–4)**
  Herbarium number: MG 222326
  Description: Monad, small; brevicolpi; amb circular; exine 3 µm thick, columellate.
  Life form: Herb.
  Geoenvironment: Slopes with *canga* vegetation over plinthosols.
***Species*: * Ichthyothere terminalis* (Spreng.) S.F.Blake (Figure 10: 5–6)**
  Herbarium number: MG 213971
  Description: Monad, medium; brevicolpi; amb circular; exine 3–3.5 µm thick, columellate.
  Life form: Herb
  Geoenvironment: Forested slopes and caves over plinthosols and ferralsols, poorly drained depressions and levels over plinthosols and histosols, doliniform lakes with organic mud sediment at the bottom.
***Species*: * Lepidaploa paraensis* (H.Rob.) H.Rob. (Figure 10: 7–8)**
  Herbarium number: MG 213957
  Description: Monad, medium; brevicolpi, echinolophate; amb circular; exine 4–6 µm thick, columellate.
  Life form: Subshrub.
  Geoenvironment: Slopes with *canga* vegetation over plinthosols, forested slopes and caves over plinthosols and ferralsols, poorly drained depressions and levels over plinthosols and histosols.
***Species*: * Monogereion carajensis* G.M.Barroso & R.M.King (Figure 10: 9–10)**
  Herbarium number: MG 213951
  Description: Monad, small; brevicolpi; amb circular; exine 3–4 µm thick, columellate.
  Life form: Herb.
  Geoenvironment: Forested slopes and caves over plinthosols and ferralsols, slopes with *canga* vegetation over plinthosols.
***Species*: * Riencourtia pedunculosa* (Rich.) Pruski (Figure 10: 11–12)**
  Herbarium number: MG 222330
  Description: Monad, medium; brevicolpi, elongated pores; amb circular; exine 2 µm thick, indistinct tectum.
  Life form: Herb.
  Geoenvironment: Slopes with *canga* vegetation over plinthosols, forested slopes and caves over plinthosols and ferralsols, poorly drained depressions and levels over plinthosols and histosols.

***Order*: Cucurbitales Juss. ex Bercht. & J.Presl**
***Family*: Begoniaceae C.Agardh**
***Species*: * Begonia guaduensis* Kunth (Figure 10: 13–14)**
  Herbarium number: MG 213986
  Description: Monad, small; amb circular; exine 0.5–1 µm thick, tectate.
  Life form: Subshrub.
  Geoenvironment: Slopes with *canga* vegetation over plinthosols.

***Order*: Lamiales Bromhead**
***Family*: Bignoniaceae Juss.**
***Species*: * Anemopaegma carajasense* A.H. Gentry ex Firetti-Leggieri (Figure 10: 15–16)**
  Collector number: Carreira et al. 3429
  Description: Monad, very large; eight colpi; amb circular; exine 7–7.5 µm thick, tectate.
  Life form: Shrub.
  Geoenvironment: Forested slopes and caves over plinthosols and ferralsols, slopes with *canga* vegetation over plinthosols.
***Species*: * Handroanthus serratifolius* (Vahl) S.Grose (Figure 10: 17–18)**
  Herbarium number: MG 69537
  Description: Monad, large; long colpi and pores, costate; amb circular; exine 1–2 µm thick, semitectate, columellate, homobrochate reticulate.
  Life form: Tree.
  Geoenvironment: Forested slopes and caves over plinthosols and ferralsols.
***Species*: * Jacaranda copaia* (Aubl.) D.Don (Figure 10: 19–20)**
  Herbarium number: MG 72382
  Description: Monad, medium to large; pores costate; amb circular; exine 1.5–2 µm thick.
  Life form: Tree.
  Geoenvironment: Forested slopes and caves over plinthosols and ferralsols.

**Figure 11:**
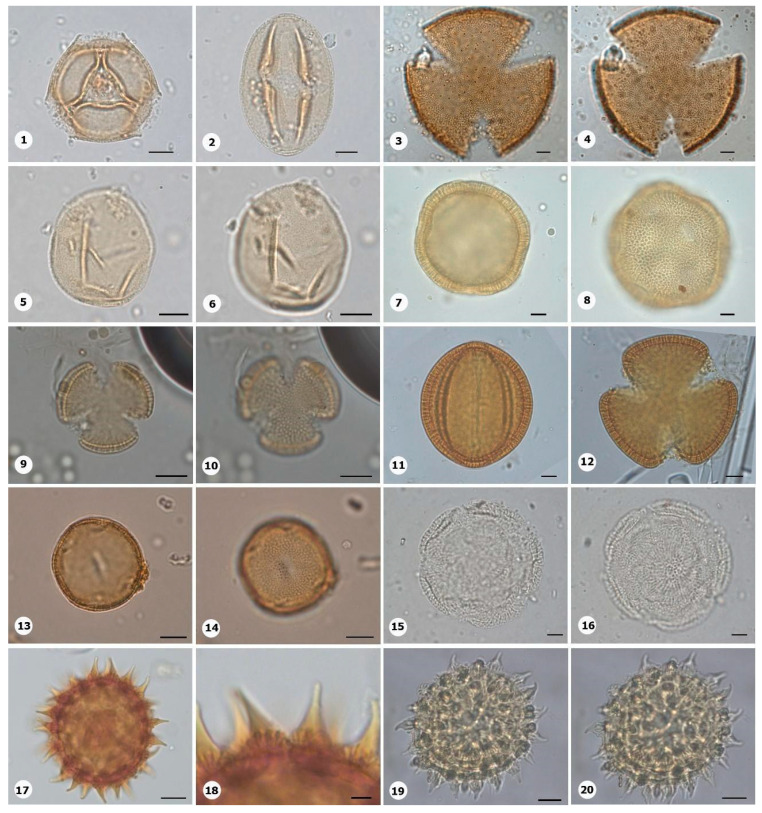
Eudicots. 1–2: *Bixa orellana*; 3–4: *Cereus hexagonus*; 5–6: *Clusia nemorosa*; 7–8: *Aniseia cernua*; 9–10: *Cuscuta insquamata*; 11–12: *Distimake macrocalyx*; 13–14: *Evolvulus filipes*; 15–16: *Evolvulus* sp. 1; 17–18: *Ipomoea asplundii*; 19–20: *Ipomoea carajasensis*; Scale: 10 µm.

***Order*: Malvales Juss.**
***Family*: Bixaceae Kunth**
***Species*: * Bixa orellana* L. (Figure 11: 1–2)**
  Herbarium number: MG 116597
  Description: Monad, large; amb triangular–obtuse–convex; exine 1.5–2 µm thick, tectate, perforate forming equatorial lobes.
  Life form: Tree.
  Geoenvironment: Forested slopes and caves over plinthosols and ferralsols.

***Order*: Caryophyllales Juss. ex Bercht. and J. Presl**
***Family*: Cactaceae Juss.**
***Species*: * Cereus hexagonus* (L.) Mill. (Figure 11: 3–4)**
  Herbarium number: HCJS 4810
  Description: Monad, very large; long colpi; amb circular; exine 2–2.2 µm thick, tectate.
  Life form: Tree and shrub.
  Geoenvironment: Slopes with *canga* vegetation over plinthosols.

***Order*: Malpighiales Juss. ex Bercht. and J. Presl**
***Family*: Clusiaceae Lindl.**
***Species*: * Clusia nemorosa* G.Mey. (Figure 11: 5–6)**
  Herbarium number: MG 213956
  Description: Monad, medium; amb circular; exine 1–1.5 µm thick, tectate.
  Life form: Tree and shrub.
  Geoenvironment: Forested slopes and caves over plinthosols and ferralsols, slopes with *canga* vegetation over plinthosols.

***Order*: Solanales Juss. ex Bercht. and J. Presl**
***Family*: Convolvulaceae Juss.**
***Species*: * Aniseia cernua* Moric. (Figure 11: 7–8)**
  Herbarium number: MG 222367
  Description: Monad, large; brevicolpi; exine 7–8 µm thick, tectate.
  Life form: Liana.
  Geoenvironment: Forested slopes and caves over plinthosols and ferralsols, slopes with *canga* vegetation over plinthosols.

***Species*: * Cuscuta insquamata* Yunck. (Figure 11: 9–10)**
  Herbarium number: MG 215432
  Description: Monad, medium; amb circular; exine 2–3 µm thick, tectate.
  Life form: Shrub, subshrub, herb.
  Geoenvironment: Forested slopes and caves over plinthosols and ferralsols, slopes with *canga* vegetation over plinthosols.
***Species*: * Distimake macrocalyx* (Ruiz and Pav.) A.R. Simões & Staples (Figure 11: 11–12)**
  Herbarium number: MG 215937
  Description: Monad, large; long colpi; amb circular; exine 4–5 µm thick, tectate.
  Life form: Liana.
  Geoenvironment: Forested slopes and caves over plinthosols and ferralsols, slopes with *canga* vegetation over plinthosols.
***Species*: * Evolvulus filipes* Mart. (Figure 11: 13–14)**
  Herbarium number: MG 215006
  Description: Monad, medium; brevicolpi; exine 1–2 µm thick, tectate.
  Life form: Herb.
  Geoenvironment: Forested slopes and caves over plinthosols and ferralsols, slopes with *canga* vegetation over plinthosols.
***Species*: * Evolvulus* sp. 1 (Figure 11: 15–16)**
  Herbarium number: MG 214054
  Description: Monad, large; five brevicolpi; amb circular; exine 4–5 µm thick, tectate.
  Life form: Shrub, subshrub, herb.
  Geoenvironment: Forested slopes and caves over plinthosols and ferralsols, slopes with *canga* vegetation over plinthosols.
***Species*: * Ipomoea asplundii* O’Donell (Figure 11: 17–18)**
  Herbarium number: MG 226331
  Description: Monad, large; large pores; exine 6 µm thick, granules are secondary in the ornamentation, conical echinae.
  Life form: Liana.
  Geoenvironment: Forested slopes and caves over plinthosols and ferralsols, slopes with *canga* vegetation over plinthosols.
***Species*: * Ipomoea carajasensis* D.F. Austin (Figure 11: 19–20)**
  Herbarium number: MG 214004
  Description: Monad, large; large pores; exine 6.5–8 µm thick, granules are secondary in the ornamentation, conical echinae.
  Life form: Liana.
  Geoenvironment: Forested slopes and caves over plinthosols and ferralsols, slopes with *canga* vegetation over plinthosols.

**Figure 12:**
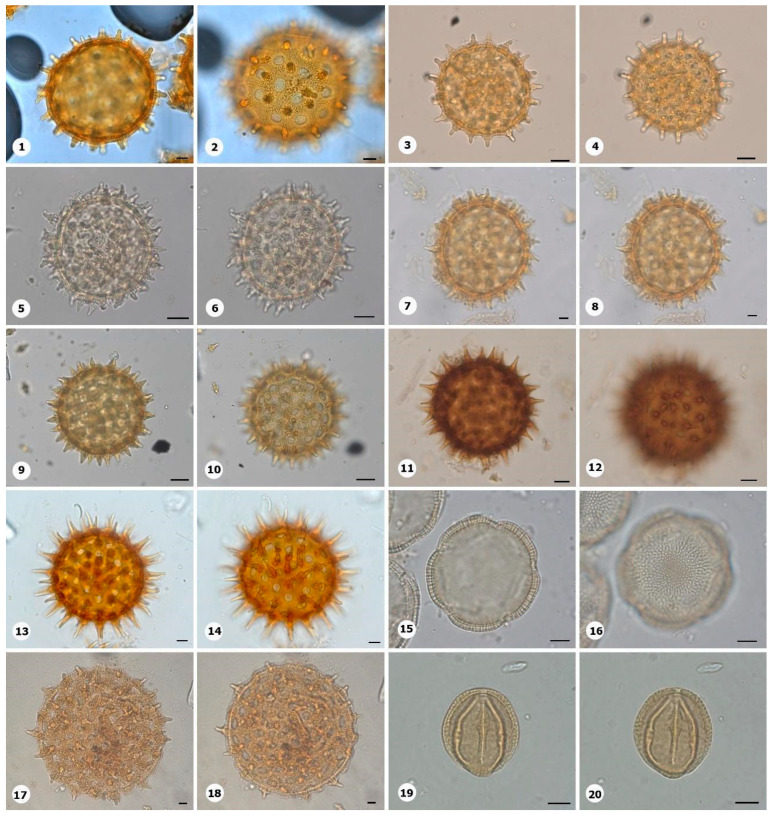
Eudicots. 1–2: *Ipomoea cavalcantei*; 3–4: *Ipomoea cavalcantei x Ipomoea marabaensis* (hibrid); 5–6: *Ipomoea decora*; 7–8: *Ipomoea goyazensis*; 9–10: *Ipomoea marabaensis* 11–12: *Ipomoea* procumbens; 13–14: *Ipomoea setifera*; 15–16: *Jacquemontia tamnifolia*; 17–18: *Turbina cordata*; 19–20: *Erythroxylum carajasense*. Scale: 10 µm.

***Species*: * Ipomoea cavalcantei* D.F. Austin (Figure 12: 1–2)**
  Herbarium number: MG 215128
  Description: Monad, large to very large; large pores; exine 4–7 µm thick, granules are secondary in the ornamentation, bulbous echinae (type 3).
  Life form: Liana.
  Geoenvironment: Slopes with *canga* vegetation over plinthosols, forested slopes and caves over plinthosols and ferralsols, poorly drained depressions and levels over plinthosols and histosols.
***Species*: * Ipomoea cavalcantei x marabaensis* (Figure 12: 3–4)**
  Herbarium number: MG 223638
  Description: Monad, large; large pores; exine 6 µm thick, granules are secondary in the ornamentation, bulbous echinae (type 2).
  Life form: Liana.
  Geoenvironment: Forested slopes and caves over plinthosols and ferralsols, slopes with *canga* vegetation over plinthosols.

***Species*: * Ipomoea decora* Meisn. (Figure 12: 5–6)**
  Herbarium number: MG 213191
  Description: Monad, large to very large; large pores; exine 8–9 µm thick, granules are secondary in the ornamentation, bulbous echinae (type 2).
  Life form: Liana.
  Geoenvironment: Forested slopes and caves over plinthosols and ferralsols, slopes with *canga* vegetation over plinthosols.
***Species*: * Ipomoea goyazensis* Gardner (Figure 12: 7–8)**
  Herbarium number: MG 227195
  Description: Monad, very large; large pores; exine 7–8 µm thick, granules are secondary in the ornamentation, bulbous echinae (type 1).
  Life form: Liana.
  Geoenvironment: Forested slopes and caves over plinthosols and ferralsols, slopes with *canga* vegetation over plinthosols.
***Species*: * Ipomoea marabaensis* D.F.Austin & Secco (Figure 12: 9–10)**
  Herbarium number: MG 227199
  Description: Monad, very large; large pores; exine 7–9 µm thick, granules are secondary in the ornamentation, bulbous echinae (type 2).
  Life form: Liana.
  Geoenvironment: Forested slopes and caves over plinthosols and ferralsols, slopes with *canga* vegetation over plinthosols.
***Species*: * Ipomoea procumbens* Mart. ex Choisy (Figure 12: 11–12)**
  Herbarium number: MG 222344
  Description: Monad, large to very large; large pores; exine 8–9 µm thick, conical echinae.
  Life form: Liana.
  Geoenvironment: Forested slopes and caves over plinthosols and ferralsols, slopes with *canga* vegetation over plinthosols.
***Species*: * Ipomoea setifera* Poir. (Figure 12: 13–14)**
  Herbarium number: MG 165270
  Description: Monad, large to very large; large pores; exine 7–8 µm thick, columellate near echinae region, conical echinae.
  Life form: Herb and liana.
  Geoenvironment: Forested slopes and caves over plinthosols and ferralsols, slopes with *canga* vegetation over plinthosols.
***Species*: * Jacquemontia tamnifolia* (L.) Griseb. (Figure 12: 15–16)**
  Herbarium number: MG 223119
  Description: Monad, large; 4–5 colporus, brevicolpi; spheroidal; exine 4–5 µm thick, columellate, tectate.
  Life form: Liana.
  Geoenvironment: Forested slopes and caves over plinthosols and ferralsols, slopes with *canga* vegetation over plinthosols.
***Species*: * Turbina cordata* (Choisy) D.F.Austin & Staples (Figure 12: 17–18)**
  Herbarium number: MG 165294
  Description: Monad, large to very large; large pores; exine 7–8 µm thick, columellate near echinae region, bulbous echinae (Type 2).
  Life form: Liana.
  Geoenvironment: Forested slopes and caves over plinthosols and ferralsols, slopes with *canga* vegetation over plinthosols.

***Order*: Malpighiales Juss. ex Bercht. & J. Presl**
***Family*: Erythroxylaceae Kunth**
***Species*: * Erythroxylum carajasense* Plowman (Figure 12: 19–20)**
  Herbarium number: MG 22354
  Description: Monad, medium; pores costate; amb circular; exine 2–3 µm thick, columellate, semitectate, heterobrochate reticulate, larger lumina in the apocolpium region.
  Life form: Shrub.
  Geoenvironment: Slopes with *canga* vegetation over plinthosols, poorly drained depressions and levels over plinthosols and histosols.

**Figure 13:**
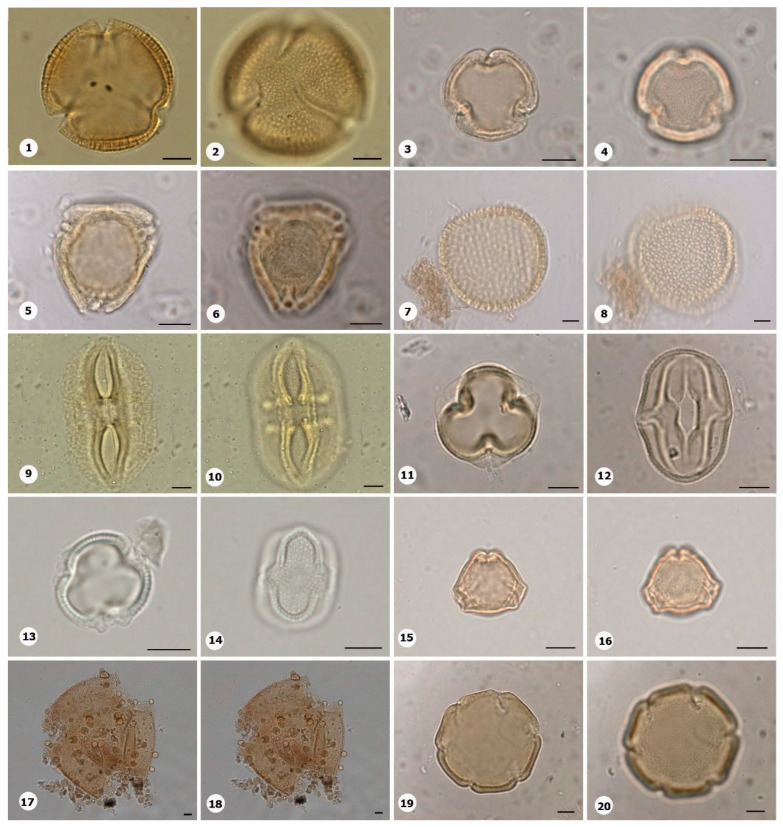
Eudicots. 1–2: *Erythroxylum nelson-rosae*; 3–4: *Alchornea discolor*; 5–6: *Aparisthmium cordatum*; 7–8: *Astraea lobata*; 9–10: *Sapium glandulosum*; 11–12: *Abrus fruticulosus*; 13–14: *Aeschynomene rudis*; 15–16: *Andira inermis*; 17–18: *Bauhinia pulchella*; 19–20: *Centrosema carajasense*. Scale: 10 µm.

***Species*: * Erythroxylum nelson-rosae* Plowman (Figure 13: 1–2)**
  Herbarium number: MG 112462
  Description: Monad, large; amb circular; exine 3–4 µm thick, columellate, tectate, columellate, heterobrochate reticulate.
  Life form: Tree and shrub.
  Geoenvironment: Slopes with *canga* vegetation over plinthosols, poorly drained depressions and levels over plinthosols and histosols.

***Family*: Euphorbiaceae Juss.**
***Species*: * Alchornea discolor* Poepp. (Figure 13: 3–4)**
  Herbarium number: MG 86323
  Description: Monad, medium; amb circular; exine 1.5–2 µm thick, tectate.
  Life form: Tree.
  Geoenvironment: Slopes with *canga* vegetation over plinthosols, poorly drained depressions and levels over plinthosols and histosols.
***Species*: * Aparisthmium cordatum* (A.Juss.) Baill. (Figure 13: 5–6)**
  Herbarium number: MG 131797
  Description: Monad, medium; operculate pores; amb triangular–obtuse–convex to straight; exine 2–2.6 µm thick, tectate.
  Life form: Tree and shrub.
  Geoenvironment: Forested slopes and caves over plinthosols and ferralsols.
***Species*: * Astraea lobata* (L.) Klotzsch (Figure 13: 7–8)**
  Herbarium number: MG 213977
  Description: Monad, large; exine 4 µm thick, columellate, capitate columellae, croton pattern.
  Life form: Shrub and herb.
  Geoenvironment: Forested slopes and caves over plinthosols and ferralsols, slopes with *canga* vegetation over plinthosols.
***Species*: * Sapium glandulosum* (L.) Morong (Figure 13: 9–10)**
  Herbarium number: MG 59033
  Description: Monad, large; lalongate endoaperture; amb triangular–obtuse–convex; exine 3–4 µm thick, columellate, tectate.
  Life form: Tree and shrub.
  Geoenvironment: Forested slopes and caves over plinthosols and ferralsols, slopes with *canga* vegetation over plinthosols.

***Order*: Fabales Bromhead**
***Family*: Fabaceae Juss.**
***Species*: * Abrus fruticulosus* Wight & Arn. (Figure 13: 11–12)**
  Herbarium number: MG 222313
  Description: Monad, medium; lalongate endoaperture; amb circular; exine 1.5–2 µm thick, columellate.
  Life form: Liana.
  Geoenvironment: Forested slopes and caves over plinthosols and ferralsols, slopes with *canga* vegetation over plinthosols.
***Species*: * Aeschynomene rudis* Benth. (Figure 13: 13–14)**
  Herbarium number: MG 222328
  Description: Monad, small; colpi with margo; amb circular, subprolate; exine 1 µm thick, columellate, tectate, heterobrochate reticulate.
  Life form: Subshrub.
  Geoenvironment: Slopes with *canga* vegetation over plinthosols, poorly drained depressions and levels over plinthosols and histosols.
***Species*: * Andira inermis* (W.Wright) DC. (Figure 13: 15–16)**
  Herbarium number: IAN 195824
  Description: Monad, small; pores annulate; amb triangular–obtuse–convex; exine 1 µm thick, tectate.
  Life form: Tree.
  Geoenvironment: Forested slopes and caves over plinthosols and ferralsols.
***Species*: * Bauhinia pulchella* Benth. (Figure 13: 17–18)**
  Herbarium number: INPA 139259
  Description: Monad, very large; long colpi, large pores; amb triangular–obtuse–convex; exine 7–8 µm thick, columellate.
  Life form: Tree and shrub.
  Geoenvironment: Poorly drained depressions and levels over plinthosols and histosols, forested slopes and caves over plinthosols and ferralsols, slopes with *canga* vegetation over plinthosols, doliniform lakes with organic mud sediments at the bottom.
***Species*: * Centrosema carajasense* Cavalcante (Figure 13: 19–20)**
  Herbarium number: MG 214006
  Description: Monad, large; six colpi, brevicolpate; amb hexangular; exine 2–3 µm thick, tectate.
  Life form: Liana.
  Geoenvironment: Slopes with *canga* vegetation over plinthosols, forested slopes and caves over plinthosols and ferralsols, poorly drained depressions and levels over plinthosols and histosols.

**Figure 14:**
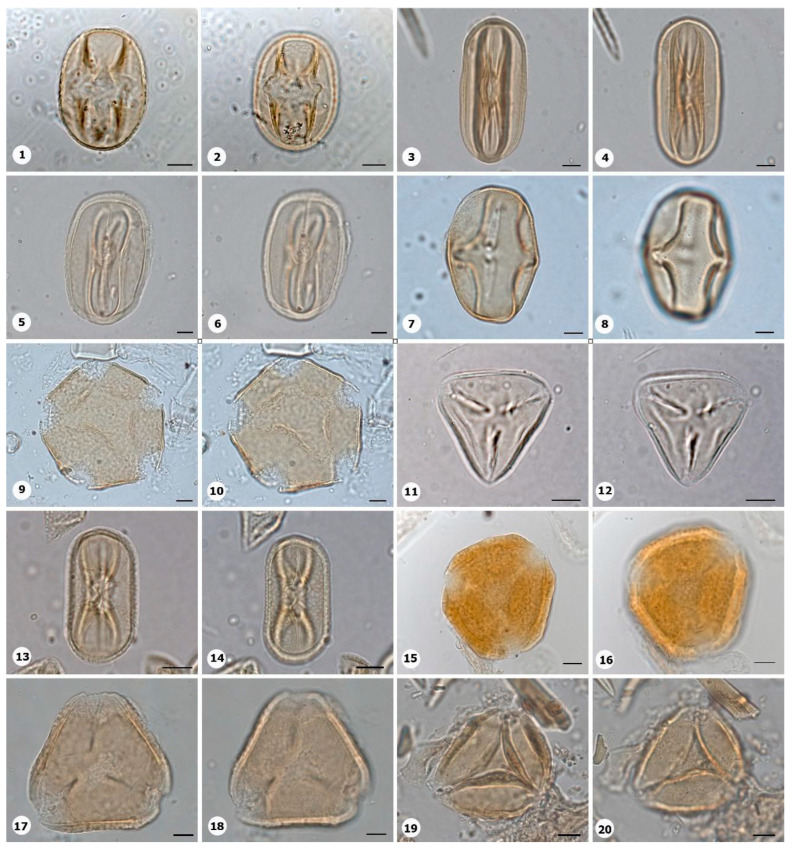
Eudicots. 1–2: *Cerradicola elliptica*; 3–4: *Chamaecrista desvauxii*; 5–6: *Chamaecrista flexuosa*; 7–8: Chamaecrista sp. 1; 9–10: *Clitoria fairchildiana*;11–12: *Copaifera martii*; 13–14: *Crotalaria maypurensis*; 15–16: *Dioclea apurensis*; 17–18: *Dioclea virgata*; 19–20: *Dipteryx odorata*. Scale: 10 µm.

***Species*: * Cerradicola elliptica* (Desv) L.P.Queiroz (Figure 14: 1–2)**
  Herbarium number: MG 36728
  Description: Monad, large; amb triangular–obtuse–convex; exine 1.5–2 µm thick, tectate, heterobrochate reticulate.
  Life form: Shrub and subshrub.
  Geoenvironment: Slopes with *canga* vegetation over plinthosols, forested slopes and caves over plinthosols and ferralsols, poorly drained depressions and levels over plinthosols and histosols.
***Species*: * Chamaecrista desvauxii* (Collad.) Killip (Figure 14: 3–4)**
  Herbarium number: MG 214058
  Description: Monad, large; pores costate; amb triangular–obtuse–convex; exine 2–2.5 µm thick, tectate.
  Life form: Shrub and subshrub.
  Geoenvironment: Slopes with *canga* vegetation over plinthosols.
***Species*: * Chamaecrista flexuosa* (L.) Greene (Figure 14: 5–6)**
  Herbarium number: MG 213980
  Description: Monad, large; pores costate; amb triangular–obtuse–convex; exine 3 µm thick, tectate.
  Life form: Shrub and subshrub.
  Geoenvironment: Slopes with *canga* vegetation over plinthosols, forested slopes and caves over plinthosols and ferralsols, poorly drained depressions and levels over plinthosols and histosols.
***Species*: * Chamaecrista* sp. 1 (Figure 14: 7–8)**
  Herbarium number: MG 214055
  Description: Monad, large; lalongate endoaperture; amb triangular–obtuse–convex; exine 2–2.5 µm thick, tectate.
  Life form: Shrub and subshrub.
  Geoenvironment: Slopes with *canga* vegetation over plinthosols, forested slopes and caves over plinthosols and ferralsols, poorly drained depressions and levels over plinthosols and histosols.

***Species*: * Clitoria fairchildiana* R.A.Howard (Figure 14: 9–10)**
  Herbarium number: IAN 195827
  Description: Monad, large; five colpi; amb circular; exine 1.5–2 µm thick, tectate.
  Life form: Tree.
  Geoenvironment: Slopes with *canga* vegetation over plinthosols.
***Species*: * Copaifera martii* Hayne (Figure 14: 11–12)**
  Herbarium number: MG 116170
  Description: Monad, medium; amb triangular–obtuse–straight; exine 1–1.5 µm thick, tectate.
  Life form: Tree and shrub.
  Geoenvironment: Forested slopes and caves over plinthosols and ferralsols, slopes with *canga* vegetation over plinthosols.
***Species*: * Crotalaria maypurensis* Kunth (Figure 14: 13–14)**
  Herbarium number: MG 222334
  Description: Monad, large; pores costate; amb triangular–obtuse–convex; exine 1.5–2 µm thick, columellate.
  Life form: Subshrub.
  Geoenvironment: Slopes with *canga* vegetation over plinthosols.
***Species*: * Dioclea apurensis* Kunth (Figure 14: 15–16)**
  Herbarium number: MG 214377
  Description: Monad, large; amb triangular–obtuse–straight; exine 5 µm thick, tectate, predominantly reticulate in the apocolpial field.
  Life form: Liana.
  Geoenvironment: Slopes with *canga* vegetation over plinthosols, forested slopes and caves over plinthosols and ferralsols, poorly drained depressions and levels over plinthosols and histosols.
***Species*: * Dioclea virgata* (Rich.) Amshoff (Figure 14: 17–18)**
  Herbarium number: MG 213961
  Description: Monad, large; amb triangular–obtuse–straight; exine 4 µm thick, tectate.
  Life form: Liana.
  Geoenvironment: Slopes with *canga* vegetation over plinthosols.
***Species*: * Dipteryx odorata* (Aubl.) Forsyth F. (Figure 14: 19–20)**
  Herbarium number: MG 33470
  Description: Monad, large, isopolar; colpi marginate; amb triangular–obtuse–convex; exine 2.5 µm thick, columellate.
  Life form: Tree.
  Geoenvironment: Forested slopes and caves over plinthosols and ferralsols.

**Figure 15:**
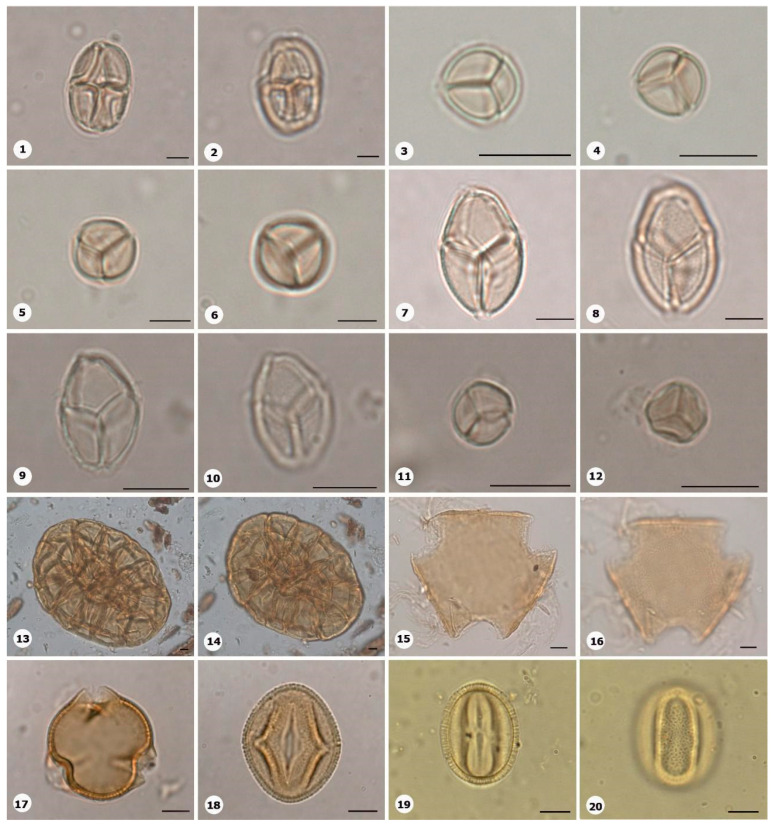
Eudicots. 1–2: *Mimosa acutistipula* var. *ferrea*; 3–4: *Mimosa aff. skinneri*; 5–6: *Mimosa carajarum*; 7–8: *Mimosa somnians* var. *viscida*; 9–10: *Mimosa xanthocentra*; 11–12: *Mimosa xanthocentra* var. mansii; 13–14: *Parkia platycephala*; 15–16: Periandra coccinea; 17–18: *Periandra mediterranea*; 19–20: *Schizolobium parahyba* var. *amazonicum*. Scale: 10 µm.

***Species*: * Mimosa acutistipula* var. *ferrea* Barneby (Figure 15: 1–2)**
  Herbarium number: MG 213962
  Description: Tetragonal tetrad, small; tetrad calymmate; exine 0.6–0.7 µm thick.
  Life form: Tree and shrub.
  Geoenvironment: Slopes with *canga* vegetation over plinthosols, forested slopes and caves over plinthosols and ferralsols, poorly drained depressions and levels over plinthosols and histosols.
***Species*: * Mimosa aff. skinneri* Benth. (Figure 15: 3–4)**
  Herbarium number: MG 222306
  Description: Tetrahedral tetrad, very small; tetrad calymmate; exine 0.5–0.8 µm thick.
  Life form: Shrub and herb.
  Geoenvironment: Poorly drained depressions and levels over plinthosols and histosols, forested slopes and caves over plinthosols and ferralsols, slopes with *canga* vegetation over plinthosols, doliniform lakes with organic mud sediments at the bottom.
***Species*: * Mimosa carajarum* (Barneby) T.P.Mendes & M.J.Silva (Figure 15: 5–6)**
  Herbarium number: MG 213978
  Description: Tetragonal tetrad, very small; tetrad calymmate; exine 0.5 µm thick.
  Life form: Shrub and herb.
  Geoenvironment: Slopes with *canga* vegetation over plinthosols, forested slopes and caves over plinthosols and ferralsols, poorly drained depressions and levels over plinthosols and histosols.

***Species*: * Mimosa somnians* var. *viscida* (Willd.) Barneby (Figure 15: 7–8)**
  Herbarium number: MG 213987
  Description: Tetragonal tetrad, small, apolar; tetrad calymmate; exine 1 µm thick.
  Life form: Shrub and subshrub.
  Geoenvironment: Slopes with *canga* vegetation over plinthosols, forested slopes and caves over plinthosols and ferralsols, poorly drained depressions and levels over plinthosols and histosols.
***Species*: * Mimosa xanthocentra* Mart. (Figure 15: 9–10)**
  Herbarium number: MG 22307
  Description: Tetragonal tetrad, small; tetrad calymmate; exine 0.5–1 µm thick.
  Life form: Shrub and subshrub.
  Geoenvironment: Slopes with *canga* vegetation over plinthosols, forested slopes and caves over plinthosols and ferralsols, poorly drained depressions and levels over plinthosols and histosols.
***Species*: * Mimosa xanthocentra* var. *mansii* (Mart.) Barneby (Figure 15: 11–12)**
  Herbarium number: MG 198053
  Description: Tetragonal tetrad, very small; tetrad calymmate; exine 0.5 µm thick.
  Life form: Subshrub.
  Geoenvironment: Slopes with *canga* vegetation over plinthosols, forested slopes and caves over plinthosols and ferralsols, poorly drained depressions and levels over plinthosols and histosols.
***Species*: * Parkia platycephala* Benth. (Figure 15: 13–14)**
  Herbarium number: MG 112372
  Description: Polyads, very large; polyads calymmate with > 20 united grains; exine 3.5–5 µm thick.
  Life form: Tree.
  Geoenvironment: Poorly drained depressions and levels over plinthosols and histosols, forested slopes and caves over plinthosols and ferralsols, slopes with *canga* vegetation over plinthosols, doliniform lakes with organic mud sediments at the bottom.
***Species*: * Periandra coccinea* (Schrad.) Benth. (Figure 15: 15–16)**
  Herbarium number: MG 99510
  Description: Monad, large; long colpi, marginate, pores large; amb triangular–obtuse–straight; exine 3 µm thick.
  Life form: Liana.
  Geoenvironment: Slopes with *canga* vegetation over plinthosols, forested slopes and caves over plinthosols and ferralsols, poorly drained depressions and levels over plinthosols and histosols.
***Species*: * Periandra mediterranea* (Vell.) Taub. (Figure 15: 17–18)**
  Herbarium number: MG 213963
  Description: Monad, medium; colpi marginate; amb triangular–obtuse–convex; exine 1.5–2 µm thick.
  Life form: Shrub and subshrub.
  Geoenvironment: Slopes with *canga* vegetation over plinthosols, forested slopes and caves over plinthosols and ferralsols, poorly drained depressions and levels over plinthosols and histosols.
***Species*: * Schizolobium parahyba* var. *amazonicum* (Huber ex Ducke) Barneby (Figure 15: 19–20)**
  Herbarium number: MG 30663
  Description: Monad, medium; colpi marginate; amb circular; exine 2–2.5 µm thick, heterobrochate reticulate.
  Life form: Tree and shrub.
  Geoenvironment: Forested slopes and caves over plinthosols and ferralsols.

**Figure 16:**
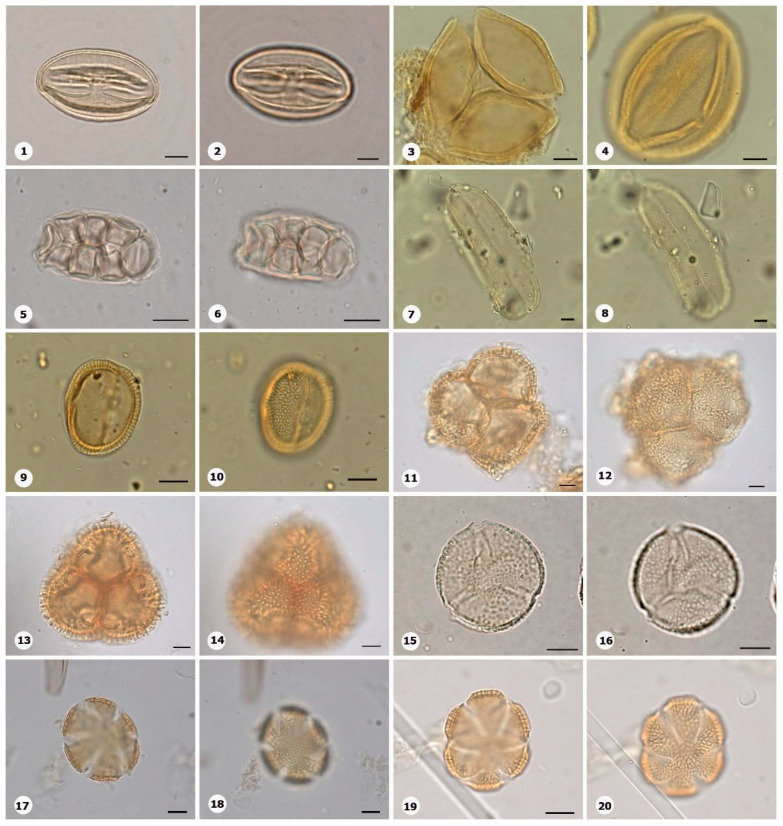
Eudicots. 1–2: *Senna multijuga*; 3–4: *Senna siamea*; 5–6: *Stryphnodendron pulcherrimum*; 7–8: *Stylosanthes humilis*; 9–10: *Tachigali vulgaris*; 11–12: *Chelonanthus purpurascens*; 13–14: *Schultesia benthamiana*; 15–16: *Vismia cayennensis*; 17–18: *Hyptis atrorubens*; 19–20: *Hyptis parkeri*. Scale: 10 µm.

***Species*: * Senna multijuga* (Rich.) H.S.Irwin & Barneby (Figure 16: 1–2)**
  Herbarium number: MG 3175
  Description: Monad, medium; pores costate; amb triangular–obtuse–convex; exine 2–2.5 µm thick.
  Life form: Tree and shrub.
  Geoenvironment: Forested slopes and caves over plinthosols and ferralsols, slopes with *canga* vegetation over plinthosols.
***Species*: * Senna siamea* (Lam.) H.S.Irwin & Barneby (Figure 16: 3–4)**
  Herbarium number: IAN 195829
  Description: Monad, medium; brevicolpi; circular; exine 2–2.5 µm thick.
  Life form: Tree.
  Geoenvironment: Exotic species, forested slopes and caves over plinthosols and ferralsols, slopes with *canga* vegetation over plinthosols.
***Species*: * Stryphnodendron pulcherrimum* (Willd.) Hochr. (Figure 16: 5–6)**
  Herbarium number: MG 147939
  Description: Polyads, medium; polyads calymmate with 16 united grains; exine 1–1.5 µm thick.
  Life form: Tree.
  Geoenvironment: Forested slopes and caves over plinthosols and ferralsols.
***Species*: * Stylosanthes humilis* Kunth (Figure 16: 7–8)**
  Herbarium number: MG 120653
  Description: Monad, large; amb elliptical; exine 2–2.5 µm thick, columellate.
  Life form: Subshrub.
  Geoenvironment: Slopes with *canga* vegetation over plinthosols.
***Species*: * Tachigali vulgaris* L.G.Silva & H.C.Lima (Figure 16: 9–10)**
  Herbarium number: MG 59069
  Description: Monad, medium; colpi slightly marginate; amb circular; exine 2–2.5 µm thick, columellate.
  Life form: Tree.
  Geoenvironment: Forested slopes and caves over plinthosols and ferralsols, slopes with *canga* vegetation over plinthosols.

***Order*: Gentianales Juss. ex Bercht. & J.Presl**
***Family*: Gentianaceae Juss.**
***Species*: * Chelonanthus purpurascens* (Aubl.) Struwe et al. (Figure 16: 11–12)**
  Herbarium number: MG 214028
  Description: Tetrahedral tetrad, large; colpi marginate, calymmate; triangular in lateral view; 4–5 µm thick, tectate, columellate.
  Life form: Tree.
  Geoenvironment: Slopes with *canga* vegetation over plinthosols.
***Species*: * Schultesia benthamiana* Klotzsch ex Griseb (Figure 16: 13–14)**
  Herbarium number: MG 214028
  Description: Tetrahedral tetrad, large; colpi marginate, calymmate; triangular in lateral view; 5–6 µm thick, tectate, columellate, curvimurate.
  Life form: Herb.
  Geoenvironment: Slopes with *canga* vegetation over plinthosols, poorly drained depressions and levels over plinthosols and histosols.

***Order*: Malpighiales Juss. ex Bercht. & J.Presl**
***Family*: Hypericaceae Juss.**
***Species*: * Vismia cayennensis* (Jacq.) Pers. (Figure 16: 15–16)**
  Herbarium number: MG 87024
  Description: Monad, medium to large; colpi marginate; amb circular; exine 1–1.5 µm thick, tectate, columellate, heterobrochate reticulate, microreticulate near margines.
  Life form: Tree.
  Geoenvironment: Forested slopes and caves over plinthosols and ferralsols.
***Order*: Lamiales Bromhead**
***Family*: Lamiaceae Lindl.**
***Species*: * Hyptis atrorubens* Poit. (Figure 16: 17–18)**
  Herbarium number: MG 87024
  Description: Monad, medium; six long colpi; amb circular; exine 2.5–3 µm thick, tectate, columellate heterobrochate reticulate.
  Life form: Herb.
  Geoenvironment: Poorly drained depressions and levels over plinthosols and histosols.
***Species*: * Hyptis parkeri* Benth. (Figure 16: 19–20)**
  Herbarium number: MG 120793
  Description: Monad, medium; six long colpi; amb circular; exine 2.5–3 µm thick, tectate, columellate, heterobrochate reticulate.
  Life form: Herb.
  Geoenvironment: Forested slopes and caves over plinthosols and ferralsols.

**Figure 17:**
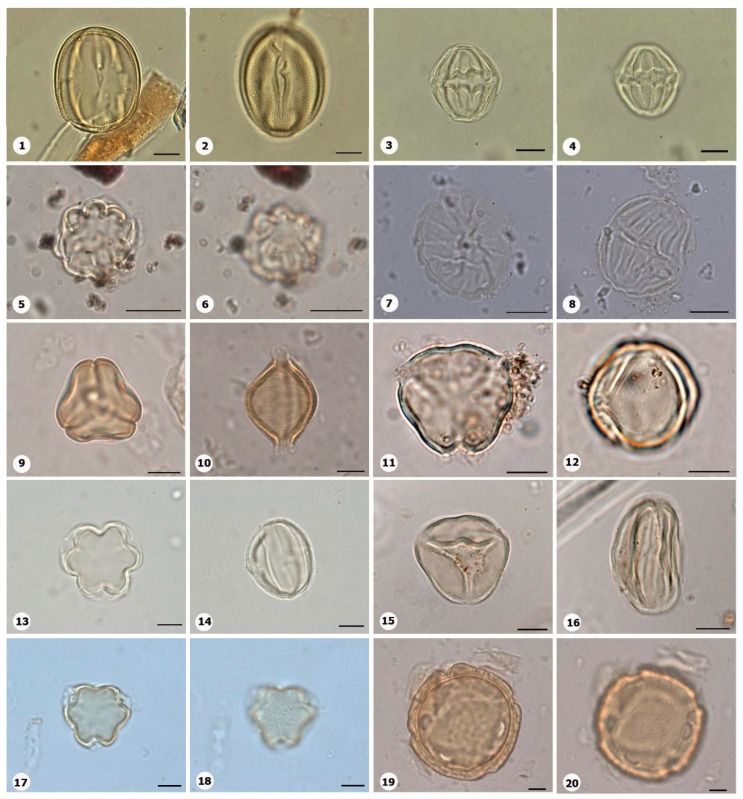
Eudicots. 1–2: *Bertholletia excelsa*; 3–4: *Utricularia pusilla*; 5–6: *Utricularia* sp. 1; 7–8: *Utricularia* sp. 2; 9–10: *Cuphea annulata*; 11–12: *Cuphea carajasensis*; 13–14: *Cuphea* sp. 1; 15–16: *Cuphea* sp. 2;17–18: *Cuphea* sp. 3; 19- 20: *Banisteriopsis appressa*. Scale: 10 µm.

***Order*: Ericales Mart.**
***Family*: Lecythidaceae A.Juss.**
***Species*: * Bertholletia excelsa* Bonpl. (Figure 17: 1–2)**
  Herbarium number: MG 60382
  Description: Monad, medium; amb triangular–obtuse–convex; exine 2 µm thick, tectate, columellate.
  Life form: Tree.
  Geoenvironment: Forested slopes and caves over plinthosols and ferralsols.

***Order*: Lamiales Bromhead**
***Family*: Lentibulariaceae Rich**
***Species*: * Utricularia pusilla* Vahl (Figure 17: 3–4)**
  Herbarium number: MG 222342
  Description: Monad, small, isopolar; 8–9 colporus; amb circular; exine 1 µm thick, tectate.
  Life form: Herb.
  Geoenvironment: Poorly drained depressions and levels over plinthosols and histosols, doliniform lakes with organic mud sediment at the bottom.
***Species*: * Utricularia* sp. 1 (Figure 17: 5–6)**
  Herbarium number: MG 213996
  Description: Monad, small; 9–10 colpi; amb circular; exine 0.5–0.7 µm thick, tectate.
  Life form: Herb.
  Geoenvironment: Poorly drained depressions and levels over plinthosols and histosols, doliniform lakes with organic mud sediment at the bottom.
***Species*: * Utricularia* sp. 2 (Figure 17: 7–8)**
  Herbarium number: MG 214072
  Description: Monad, medium; 13–15 colporus; amb circular; exine 1.5–2 µm thick, tectate.
  Life form: Herb.
  Geoenvironment: Poorly drained depressions and levels over plinthosols and histosols, doliniform lakes with organic mud sediment at the bottom.

***Order*: Myrtales Juss. ex Bercht. & J.Presl**
***Family*: Lythraceae J.St.-Hil.**
***Species*: * Cuphea annulata* Koehne (Figure 17: 9–10)**
  Herbarium number: MG 213965
  Description: Monad, medium; short colpi, protruding pores; amb triangular–obtuse–straight; exine 1–3 µm thick, thickening in the region between the endoapertures, tectate.
  Life form: Shrub and subshrub.
  Geoenvironment: Slopes with *canga* vegetation over plinthosols, poorly drained depressions and levels over plinthosols and histosols.

***Species*: * Cuphea carajasensis* Lourteig (Figure 17: 11–12)**
  Herbarium number: MG 222339
  Description: Monad, medium; short colpi, protruding and large pores with 4–5 µm diameter; amb triangular–obtuse–straight; exine 1–3 µm thick, thickening in the region between the endoapertures, tectate.
  Life form: Shrub and subshrub.
  Geoenvironment: Slopes with *canga* vegetation over plinthosols, forested slopes and caves over plinthosols and ferralsols, poorly drained depressions and levels over plinthosols and histosols.
***Species*: * Cuphea* sp. 1 (Figure 17: 13–14)**
  Herbarium number: MG 214023
  Description: Monad, medium; short colpi, protruding pores; amb triangular–obtuse–straight; exine 1–3 µm thick, thickening in the region between the endoapertures, tectate.
  Life form: Shrub, subshrub, herb.
  Geoenvironment: Slopes with *canga* vegetation over plinthosols, forested slopes and caves over plinthosols and ferralsols, poorly drained depressions and levels over plinthosols and histosols.
***Species*: * Cuphea* sp. 2 (Figure 17: 15–16)**
  Herbarium number: MG 214065
  Description: Monad, small; colpi marginate; amb triangular–obtuse–convex; exine 1–3 µm thick.
  Life form: Shrub, subshrub, herb.
  Geoenvironment: Slopes with *canga* vegetation over plinthosols, forested slopes and caves over plinthosols and ferralsols, poorly drained depressions and levels over plinthosols and histosols.
***Species*: * Cuphea* sp. 3 (Figure 17: 17–18)**
  Herbarium number: MG 213967
  Description: Monad, medium; brevicolpi, protruding pores; amb triangular–obtuse–concave; exine 1–2 µm thick, tectate.
  Life form: Shrub, subshrub, herb.
  Geoenvironment: Slopes with *canga* vegetation over plinthosols, forested slopes and caves over plinthosols and ferralsols, poorly drained depressions and levels over plinthosols and histosols.

***Order*: Malpighiales Juss. ex Bercht. & J.Presl**
***Family*: Malpighiaceae Juss.**
***Species*: * Banisteriopsis appressa* (B.Gates) R.F.Almeida & M.Pell. (Figure 17: 19–20)**
  Herbarium number: MG 213959
  Description: Monad, large; large pores, pseudocolpate; exine 3–4 µm thick, tectate.
  Life form: Shrub, subshrub, liana.
  Geoenvironment: Slopes with *canga* vegetation over plinthosols.

**Figure 18:**
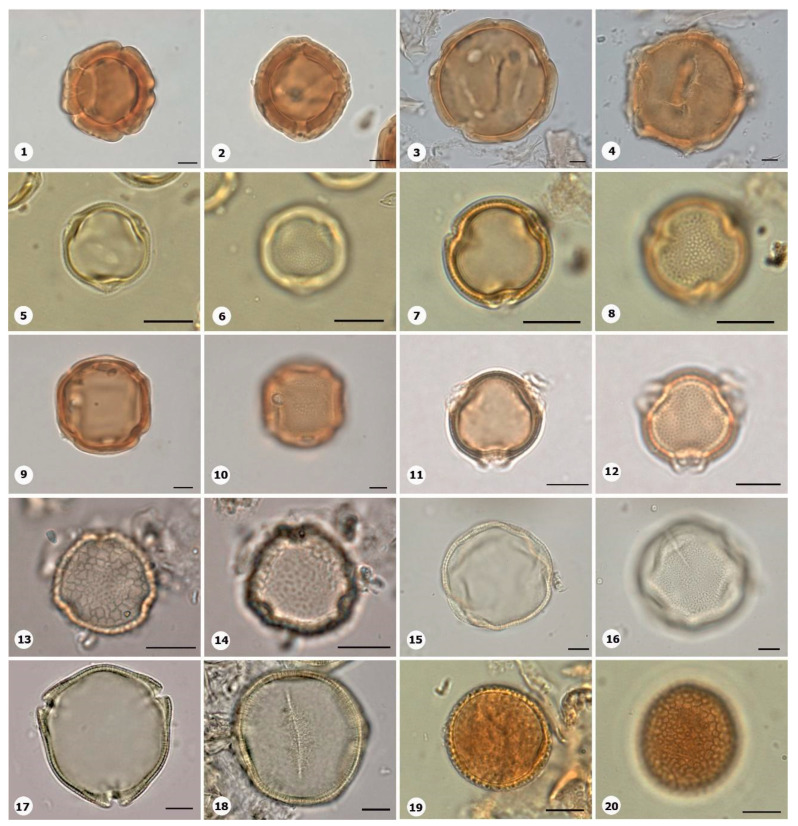
Eudicots. 1–2: *Banisteriopsis malifolia*; 3–4: *Banisteriopsis* sp. 1; 5–6: *Byrsonima chrysophylla*; 7–8: *Byrsonima spicata*; 9–10: *Diplopterys pubipetala*; 11–12: *Spachea lactescens*; 13–14: *Guazuma* ulmifolia; 15–16: *Melochia arenosa*; 17–18: *Melochia spicata*; 19–20: *Theobroma grandiflorum*. Scale: 10 µm.

***Species*: * Banisteriopsis malifolia* (Nees & Mart.) B.Gates (Figure 18: 1–2)**
  Herbarium number: MG 222352
  Description: Monad, large; large pores, pseudocolpate; exine 8–9 µm thick, tectate.
  Life form: Shrub and subshrub.
  Geoenvironment: Forested slopes and caves over plinthosols and ferralsols, slopes with *canga* vegetation over plinthosols.
***Species*: * Banisteriopsis* sp. 1 C.B.Rob. ex Small (Figure 18: 3–4)**
  Herbarium number: MG 214076

Description: Monad, large; large pores, pseudocolpate; exine 5–6 µm thick, tectate.
  Life form: Shrub, subshrub, liana.
  Geoenvironment: Forested slopes and caves over plinthosols and ferralsols, slopes with *canga* vegetation over plinthosols.
***Species*: * Byrsonima chrysophylla* Kunth (Figure 18: 5–6)**
  Herbarium number: MG 125695
  Description: Monad, small; protruding pores, brevicolpi; amb circular; exine 1 µm thick, tectate, columellate.
  Life form: Tree and shrub.
  Geoenvironment: Poorly drained depressions and levels over plinthosols and histosols.
***Species*: * Byrsonima spicata* (Cav.) DC. (Figure 18: 7–8)**
  Herbarium number: IAN 195825
  Description: Monad, small; protruding pores, brevicolpi; amb circular; exine 1 µm thick, tectate, columellate.
  Life form: Tree.
  Geoenvironment: Slopes with *canga* vegetation over plinthosols.
***Species*: * Diplopterys pubipetala* (A.Juss.) W.R.Anderson & C.C.Davis (Figure 18: 9–10)**
  Herbarium number: MG 85808
  Description: Monad, large; pantoporate, large pores, pseudocolpate; exine 5–6 µm thick, tectate.
  Life form: Liana.
  Geoenvironment: Forested slopes and caves over plinthosols and ferralsols, slopes with *canga* vegetation over plinthosols.
***Species*: * Spachea lactescens* (Ducke) R.F.Almeida & M.Pell. (Figure 18: 11–12)**
  Herbarium number: MG 30032
  Description: Monad, small; protruding pores, brevicolpi; amb circular; exine 1.5–1.7 µm thick, tectate, columellate.
  Life form: Tree.
  Geoenvironment: Forested slopes and caves over plinthosols and ferralsols.

***Order*: Malvales Juss.**
***Family*: Malvaceae Juss.**
***Species*: * Guazuma ulmifolia* Lam. (Figure 18: 13–14)**
  Herbarium number: MG 30109
  Description: Monad, small; amb circular; exine 1.5 µm thick, tectate, heterobrochate reticulate.
  Life form: Tree.
  Geoenvironment: Slopes with *canga* vegetation over plinthosols.
***Species*: * Melochia arenosa* Benth. (Figure 18: 15–16)**
  Herbarium number: MG 222336
  Description: Monad, large; brevicolpi, lalongate endoaperture; amb circular; exine 2–3 µm thick, tectate, columellate.
  Life form: Tree and shrub.
  Geoenvironment: Poorly drained depressions and levels over plinthosols and histosols.
***Species*: * Melochia spicata* (L.) Fryxell (Figure 18: 17–18)**
  Herbarium number: HCJS 023
  Description: Monad, medium to large; brevicolpi, protruding pores; amb triangular–obtuse–convex; exine 2–3 µm thick, tectate, columellate, reticulate near the apertural region.
  Life form: Tree and shrub.
  Geoenvironment: Poorly drained depressions and levels over plinthosols and histosols.
***Species*: * Theobroma grandiflorum* (Willd. ex Spreng.) K.Schum. (Figure 18: 19–20)**
  Herbarium number: IAN 195841
  Description: Monad, small to medium; brevicolpi, lalongate endoaperture; amb circular; exine 1–1.5 µm thick, tectate, columellate, heterobrochate reticulate.
  Life form: Tree.
  Geoenvironment: Forested slopes and caves over plinthosols and ferralsols.

**Figure 19:**
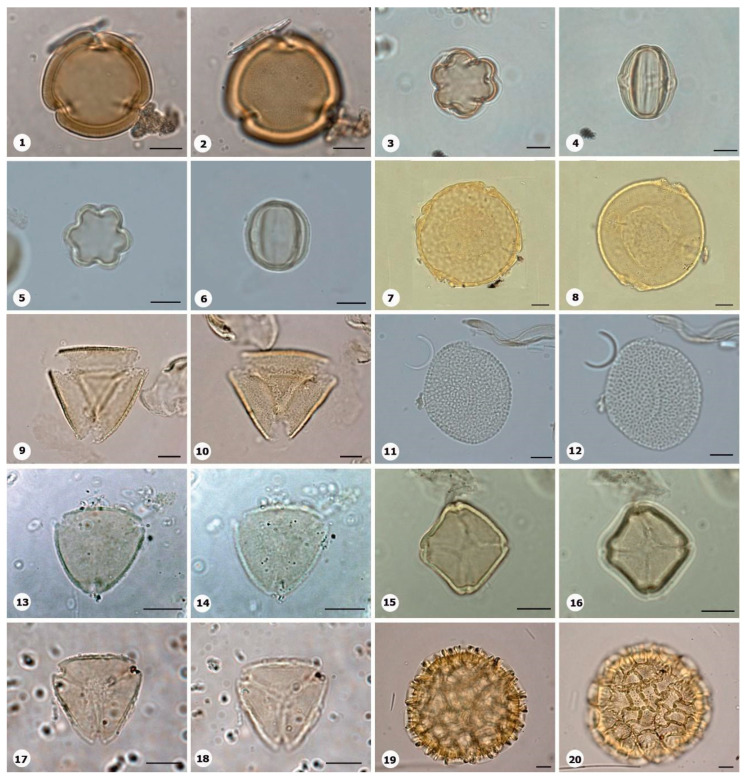
Eudicots. 1–2: *Norantea guianensis*; 3–4: *Miconia chamissois*; 5–6: *Pleroma stenocarpum*; 7–8: *Carapa guianensis*; 9–10: *Nymphoides humboldtiana*; 11–12: *Virola michelii*; 13–14: *Eugenia flavescens*; 15–16: *Eugenia punicifolia*; 17–18: *Myrcia multiflora*; 19–20: *Passiflora glandulosa*. Scale: 10 µm.

***Order*: Ericales Bercht. & J.Presl**
***Family*: Marcgraviaceae Bercht. & J.Presl**
***Species*: * Norantea guianensis* Aubl. (Figure 19: 1–2)**
  Herbarium number: MG 123123
  Description: Monad, medium; brevicolpi; amb circular; exine 3.5–4 µm thick, tectate, columellate.
  Life form: Liana.
  Geoenvironment: Slopes with *canga* vegetation over plinthosols.

***Order*: Myrtales Juss. ex Bercht. & J.Presl**
***Family*: Melastomataceae Juss.**
***Species*: * Miconia chamissois* Naudin (Figure 19: 3–4)**
  Herbarium number: MG 214002
  Description: Monad, medium; pseudocolpi, lalongate endoaperture; amb circular; exine 2 µm thick, tectate.
  Life form: Tree and shrub.
  Geoenvironment: Forested slopes and caves over plinthosols and ferralsols, slopes with *canga* vegetation over plinthosols.
***Species*: * Pleroma stenocarpum* (Schrank et Mart. ex DC.) Triana (Figure 19: 5–6)**
  Herbarium number: MG 214035
  Description: Monad, small; pores slightly costate, three pseudocolpi; amb circular; exine 1–1.5 µm thick, tectate.
  Life form: Tree.
  Geoenvironment: Forested slopes and caves over plinthosols and ferralsols, slopes with *canga* vegetation over plinthosols.

***Order*: Sapindales Juss. ex Bercht. & J.Presl**
***Family*: Meliaceae Juss.**
***Species*: * Carapa guianensis* Aubl. (Figure 19: 7–8)**
  Herbarium number: MG 77934
  Description: Monad, large; four brevicolpi, marginate; amb circular; exine 2–4 µm thick, tectate, columellate.
  Life form: Tree.
  Geoenvironment: Managed species, forested slopes and caves over plinthosols and ferralsols.

***Order*: Asterales Link**
***Family*: Menyanthaceae Dumort.**
**Species: *Nymphoides humboldtiana* (Kunth) Kuntze (Figure 19: 9–10)**
  Herbarium number: MG 214022
  Description: Monad, small; large colpi, marginate; amb triangular–obtuse–straight; exine 2 µm thick, tectate, columellate.
  Life form: Herb.
  Geoenvironment: Doliniform lakes with organic mud sediments at the bottom.

***Order*: Magnoliales Bromhead**
***Family*: Myristicaceae R.Br.**
***Species*: * Virola michelii* Heckel (Figure 19: 11–12)**
  Herbarium number: IAN 146780
  Description: Monad, medium; large colpi, marginate; amb triangular–obtuse–straight; exine 1.5–2 µm thick, tectate, columellate, heterobrochate reticulate.
  Life form: Tree.
  Geoenvironment: Forested slopes and caves over plinthosols and ferralsols.

***Order*: Myrtales Juss. ex Bercht. & J. Presl**
***Family*: Myrtaceae Juss.**
***Species*: * Eugenia flavescens DC.* (Figure 19: 13–14)**
  Herbarium number: MG 112463
  Description: Monad, small; three colpi; amb triangular–obtuse–convex; exine 1 µm thick, tectate.
  Life form: Tree and shrub.
  Geoenvironment: Forested slopes and caves over plinthosols and ferralsols, slopes with *canga* vegetation over plinthosols.
***Species*: * Eugenia punicifolia* (Kunth) DC. (Figure 19: 15–16)**
  Herbarium number: MG 174698
  Description: Monad, small to medium; 3–4 colpi; amb triangular–obtuse–concave to quadrangular; exine 1 µm thick, tectate.
  Life form: Tree and shrub.
  Geoenvironment: Forested slopes and caves over plinthosols and ferralsols, slopes with *canga* vegetation over plinthosols.
***Species*: * Myrcia multiflora* (Lam.) DC. (Figure 19: 17–18)**
  Herbarium number: MG 112459
  Description: Monad, small; three colpi; amb triangular–obtuse–straight; exine 1.5–2 µm thick, tectate.
  Life form: Tree and shrub.
  Geoenvironment: Slopes with *canga* vegetation over plinthosols, forested slopes and caves over plinthosols and ferralsols, poorly drained depressions and levels over plinthosols and histosols.

***Family*: Passifloraceae Juss. ex Roussel**
***Species*: * Passiflora glandulosa Cav.* (Figure 19: 19–20)**
  Herbarium number: MG 216102
  Description: Monad, large; 3-mesocolpi fused in pairs; amb circular; exine 9–10 µm thick, tectate, columellate, curvimurate, sometimes bacula are observed.
  Life form: Liana.
  Geoenvironment: Forested slopes and caves over plinthosols and ferralsols, slopes with *canga* vegetation over plinthosols.

**Figure 20:**
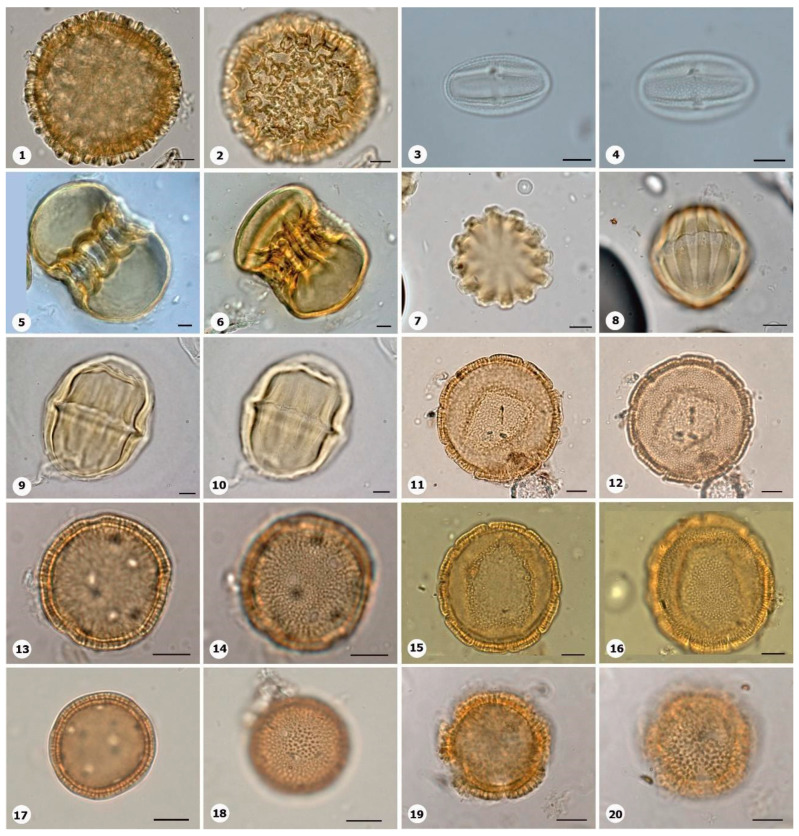
Eudicots. 1–2: *Passiflora tholozanii*; 3–4: *Phyllanthus hyssopifolioides*; 5–6: *Caamembeca spectabilis*; 7–8: *Securidaca diversifolia*; 9–10: *Senega adenophora*; 11–12: *Borreria alata*; 13–14: *Borreria elaiosulcata*; 15–16: *Borreria latifolia*; 17–18: *Borreria paraensis*; 19–20: *Carajasia cangae*. Scale: 10 µm.

***Species*: * Passiflora tholozanii* Sacco (Figure 20: 1–2)**
  Herbarium number: MG 216123
  Description: Monad, large; 3-mesocolpi fused in pairs; amb circular; exine 8–9 µm thick, tectate, columellate, curvimurate, sometimes bacula are observed.
  Life form: Liana.
  Geoenvironment: Forested slopes and caves over plinthosols and ferralsols, slopes with *canga* vegetation over plinthosols.

***Family*: Phyllanthaceae Martinov**
***Species*: * Phyllanthus hyssopifolioides* Kunth (Figure 20: 3–4)**
  Herbarium number: MG 216123
  Description: Monad, medium; pores costate, marginate; amb elliptical; exine 1 µm thick, tectate, columellate, heterobrochate reticulate.
  Life form: Shrub and herb.
  Geoenvironment: Poorly drained depressions and levels over plinthosols and histosols.

***Order*: Fabales Bromhead**
***Family*: Polygalaceae Hoffmanns. & Link**
***Species*: * Caamembeca spectabilis* (DC.) J.F.B.Pastore (Figure 20: 5–6)**
  Herbarium number: MG 222363
  Description: Monad, large to very large; 12 colporus, zonorate; amb elliptical; exine 4–5 µm thick, tectate.
  Life form: Subshrub.
  Geoenvironment: Slopes with *canga* vegetation over plinthosols, poorly drained depressions and levels over plinthosols and histosols.

***Species*: * Securidaca diversifolia* (L.) S.F.Blake (Figure 20: 7–8)**
  Herbarium number: MG 222364
  Description: Monad, medium; 12 colporus, zonorate; amb elliptical; exine 2–3 µm thick, tectate.
  Life form: Liana.
  Geoenvironment: Slopes with *canga* vegetation over plinthosols, poorly drained depressions and levels over plinthosols and histosols.
***Species*: * Senega adenophora* (DC.) J.F.B.Pastore (Figure 20: 9–10)**
  Herbarium number: MG 222337
  Description: Monad, large; 12 colporus, zonorate; amb elliptical; exine 3–4 µm thick, tectate.
  Life form: Herb.
  Geoenvironment: Slopes with *canga* vegetation over plinthosols, poorly drained depressions and levels over plinthosols and histosols.

***Order*: Gentianales Juss. ex Bercht. & J. Presl**
***Family*: Rubiaceae Juss.**
***Species*: * Borreria alata* (Aubl.) DC. (Figure 20: 11–12)**
  Herbarium number: MG 115799
  Description: Monad, medium; exine 3–3.5 µm thick, tectate, columellate.
  Life form: Subshrub.
  Geoenvironment: Slopes with *canga* vegetation over plinthosols, poorly drained depressions and levels over plinthosols and histosols.
***Species*: * Borreria elaiosulcata* E.L.Cabral & L.M.Miguel (Figure 20: 13–14)**
  Herbarium number: MG 222332
  Description: Monad, medium; exine 2–3 µm thick, tectate, columellate.
  Life form: Subshrub.
  Geoenvironment: Poorly drained depressions and levels over plinthosols and histosols, forested slopes and caves over plinthosols and ferralsols, slopes with *canga* vegetation over plinthosols, doliniform lakes with organic mud sediments at the bottom.
***Species*: * Borreria latifolia* (Aubl.) K.Schum (Figure 20: 15–16)**
  Herbarium number: MG 115799
  Description: Monad, large; exine 4 µm thick, tectate, columellate.
  Life form: Shrub and herb.
  Geoenvironment: Poorly drained depressions and levels over plinthosols and histosols.
***Species*: * Borreria paraensis* E.L.Cabral & Bacigalupo (Figure 20: 17–18)**
  Herbarium number: MG 214083
  Description: Monad, small; exine 2–2.5 µm thick, tectate, columellate.
  Life form: Shrub and herb.
  Geoenvironment: Slopes with *canga* vegetation over plinthosols, forested slopes and caves over plinthosols and ferralsols, poorly drained depressions and levels over plinthosols and histosols.
***Species*: * Carajasia cangae* R.M.Salas, E.L.Cabral & Dessein (Figure 20: 19–20)**
  Herbarium number: MG 213972
  Description: Monad, medium; 5–7 brevicolpi; exine 2 µm thick, tectate, columellate.
  Life form: Herb.
  Geoenvironment: Slopes with *canga* vegetation over plinthosols, poorly drained depressions and levels over plinthosols and histosols.

**Figure 21:**
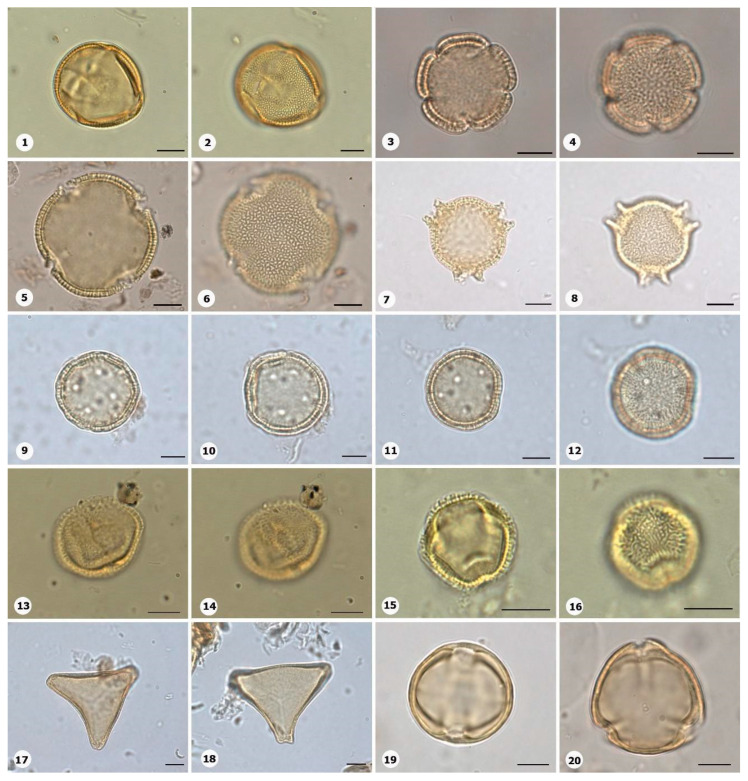
Eudicots. 1–2: *Ixora coccinea*; 3–4: *Mitracarpus carajasensis*; 5–6: *Perama carajensis*; 7–8: *Perama* sp. 1; 9–10: *Spermacoce* sp. 1; 11–12: *Spermacoce* sp. 2; 13–14: *Pilocarpus microphyllus*; 15–16: *Zanthoxylum gardneri*; 17–18: *Serjania caracasana*; 19–20: *Solanum crinitum*. Scale: 10 µm.

***Species*: * Ixora coccinea* L. (Figure 21: 1–2)**
  Herbarium number: IAN 195851
  Description: Monad, medium; lalongate endoaperture, colpi slightly marginate; amb circular; exine 2 µm thick, tectate, columellate, heterobrochate reticulate.
  Life form: Shrub.
  Geoenvironment: Anthropic areas.
***Species*: * Mitracarpus carajasensis* E.L.Cabral, Sobrado & E.B.Souza (Figure 21: 3–4)**
  Herbarium number: MG 208513
  Description: Monad, small; five brevicolpi; amb circular; exine 2–3 µm thick, tectate, columellate.
  Life form: Herb.
  Geoenvironment: Slopes with *canga* vegetation over plinthosols, doliniform lakes with organic mud sediments at the bottom.

***Species*: * Perama carajensis* J.H.Kirkbr. (Figure 21: 5–6)**
  Herbarium number: MG 222331
  Description: Monad, medium; 4–5 brevicolpi; amb circular; exine 3 µm thick, tectate, columellate.
  Life form: Herb.
  Geoenvironment: Slopes with *canga* vegetation over plinthosols, poorly drained depressions and levels over plinthosols and histosols.
***Species*: * Perama* sp. 1 (Figure 21: 7–8)**
  Collection number: ITV 2116
  Description: Monad, medium; brevicolpi, protruding large pores; amb circular; exine 3 µm thick, tectate, columellate.
  Life form: Herb.
  Geoenvironment: Slopes with *canga* vegetation over plinthosols, poorly drained depressions and levels over plinthosols and histosols.
***Species*: * Spermacoce* sp. 1 (Figure 21: 9–10)**
  Herbarium number: MG 214024
  Description: Monad, medium; exine 2.5–3 µm thick, tectate, columellate.
  Life form: Shrub, subshrub, herb.
  Geoenvironment: Doliniform lakes with organic mud sediments at the bottom.
***Species*: * Spermacoce* sp. 2 (Figure 21: 11–12)**
  Herbarium number: MG 222338
  Description: Monad, medium; exine 2.5 µm thick, tectate, columellate.
  Life form: Shrub, subshrub, herb.
  Geoenvironment: Slopes with *canga* vegetation over plinthosols.

**Order: Sapindales Juss. ex Bercht. & J. Presl**
***Family*: Rutaceae Juss.**
***Species*: * Pilocarpus microphyllus* Stapf ex Wardlew. (Figure 21: 13–14)**
  Herbarium number: MG 37903
  Description: Monad, medium; amb elliptical; exine 2 µm thick, columellate, heterobrochate reticulate, sometimes curvimurate.
  Life form: Tree.
  Geoenvironment: Forested slopes and caves over plinthosols and ferralsols.
***Species*: * Zanthoxylum gardneri* Engl. (Figure 21: 15–16)**
  Herbarium number: MG 222372
  Description: Monad, medium; pores costate; amb elliptical; exine 2 µm thick, columellate, heterobrochate reticulate.
  Life form: Tree.
  Geoenvironment: Forested slopes and caves over plinthosols and ferralsols.

***Family*: Sapindaceae Juss.**
***Species*: * Serjania caracasana* (Jacq.) Willd. (Figure 21: 17–18)**
  Herbarium number: MG 214067
  Description: Monad, medium; longicolpi, likely lolongate; amb triangular–acute–straight to concave; exine 1.5 µm thick, thickening towards the apertural region, tectate, columellate.
  Life form: Liana.
  Geoenvironment: Forested slopes and caves over plinthosols and ferralsols.

***Order*: Solanales Juss. ex Bercht. & J. Presl**
***Family*: Solanaceae Adans.**
***Species*: * Solanum crinitum* Lam. (Figure 21: 19–20)**
  Herbarium number: MG 115832
  Description: Monad, medium; colpi marginate, slightly protruding pores costate; amb circular; exine 2 µm thick, tectate.
  Life form: Tree and shrub.
  Geoenvironment: Forested slopes and caves over plinthosols and ferralsols, slopes with *canga* vegetation over plinthosols.

**Figure 22:**
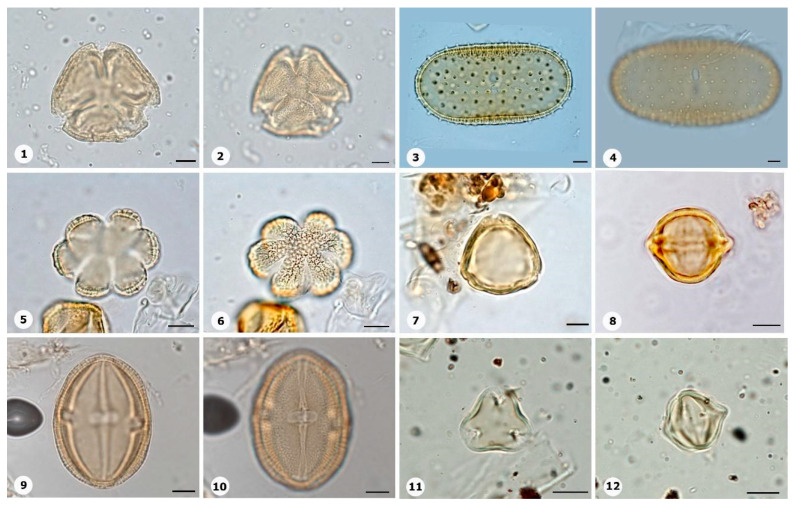
Eudicots. 1–2: *Styrax ferrugineus*; 3–4: *Turnera glaziovi*; 5–6: *Latana* sp. 1; 7–8: *Lippia grata*; 9–10: *Cissus erosa*; 11–12: *Callisthene microphylla*. Scale: 10 µm.

***Order*: Ericales Bercht. & J. Presl**
***Family*: Styracaceae DC. & Spreng.**
***Species*: * Styrax ferrugineus* Nees & Mart (Figure 22: 1–2)**
  Herbarium number: MG 213964
  Description: Monad, large; large pores; amb triangular–obtuse–straight to convex; exine 3.5–4 µm thick, tectate, columellate.
  Life form: Tree and shrub.
  Geoenvironment: Forested slopes and caves over plinthosols and ferralsols, slopes with *canga* vegetation over plinthosols.

***Order*: Malpighiales**
***Family*: Turneraceae Kunth ex DC.**
***Species*: * Turnera glaziovii* Urb. (Figure 22: 3–4)**
  Herbarium number: MG 222359
  Description: Monad, very large; brevicolpi, large elliptical pores; amb elliptical; exine 4.5–5 µm thick, thickening towards the distal face, tectate, columellate, microreticulate in distal face, regularly microechinate.
  Life form: Shrub.
  Geoenvironment: Forested slopes and caves over plinthosols and ferralsols, slopes with *canga* vegetation over plinthosols.

***Order*: Lamiales Bromhead**
***Family*: Verbenaceae J.St.-Hil.**
***Species*: * Lantana* sp. 1 (Figure 22: 5–6)**
  Herbarium number: MG 222359
  Description: Monad, medium; six colpi; amb circular; exine 3.5 µm thick, tectate, columellate, heterobrochate reticulate, sometimes curvimurate.
  Life form: Shrub, subshrub, herb.
  Geoenvironment: Forested slopes and caves over plinthosols and ferralsols, slopes with *canga* vegetation over plinthosols.
***Species*: * Lippia grata* Schauer (Figure 22: 7–8)**
  Herbarium number: MG 213955
  Description: Monad, medium; brevicolpi, marginate, lalongate; amb triangular–obtuse–straight to convex; exine 2–2.5 µm thick, tectate.
  Life form: Shrub.
  Geoenvironment: Slopes with *canga* vegetation over plinthosols.

***Order: Vitales Juss.* ex Bercht. & J. Presl**
***Family*: Vitaceae Juss.**
***Species*: * Cissus erosa* Rich. (Figure 22: 9–10)**
  Herbarium number: MG 214070
  Description: Monad, large; lalongate endoaperture, large pores costate; amb elliptical; exine 2.3–3.5 µm thick, tectate, columellate.
  Life form: Shrub and liana.
  Geoenvironment: Slopes with *canga* vegetation over plinthosols, forested slopes and caves over plinthosols and ferralsols, poorly drained depressions and levels over plinthosols and histosols.

**Order: Myrtales Juss. ex Bercht. & J. Presl**
**Family: Vochysiaceae A.St.-Hil.**
***Species*: * Callisthene microphylla* Warm. (Figure 22: 11–12)**
  Collection number: ITV 2253
  Description: Monad, small; colpi slightly marginate; amb circular; exine 1 µm thick.
  Life form: Tree and shrub.
  Geoenvironment: Forested slopes and caves over plinthosols and ferralsols, slopes with *canga* vegetation over plinthosols.

## 4. Discussion

The palynology of the *canga* vegetation of Carajás offers valuable insights into the region’s floral diversity and ecological processes. The pollen atlas reveals the dominance of eudicots (130 species) compared to monocots (62 species). This distribution aligns with the high diversity of flowering plants in the Amazonian ecosystem, emphasizing the ecological importance of eudicots, which often dominate terrestrial vegetation in terms of species richness and functional roles [49].

**Figure 23:**
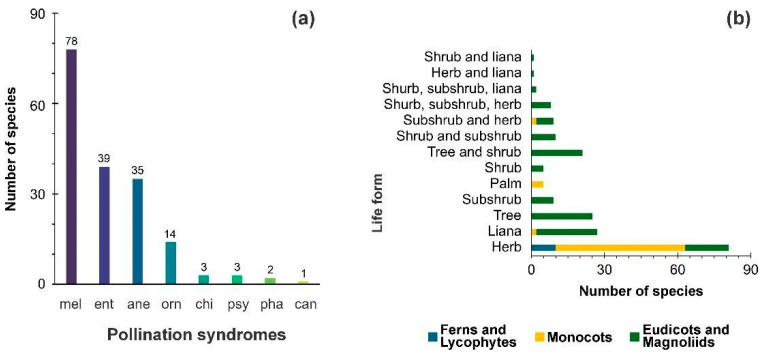
Overview of key ecological characteristics of *canga* flora: (**a**) Distribution of pollination syndromes (mel: melittophily; ane: anemophily; ent: entomophily; orn: ornithophily; chi: chiropterophily; psy: psychophily; pha: phanelophily; and can: cantharophily) among plant species in *canga* vegetation; (**b**) proportions of life forms (tree/shrub, subshrub, palm, liana, and herb) within monocotyledons and eudicotyledons in *canga* flora.

Among the pollination syndromes, melittophily (bee pollination) was the most prevalent (78 species), followed by entomophily (general insect pollination, 39 species), and anemophily (wind pollination, 35 species) (Figure 23a). These data highlight the significant role of insects, particularly bees, as primary pollinators in this environment. Less common syndromes, such as ornithophily (bird pollination, 14 species), chiropterophily (bat pollination, 3 species), and specialized insect pollination syndromes (e.g., psychophily, phanelophily, and cantharophily, ≤3 species), indicate niche adaptations that sustain the biodiversity in the ironstone habitats.

The flora of the *canga* vegetation is categorized into six types of life forms: trees, shrubs, subshrubs, palms, lianas, and herbs. Herbaceous plants are the most abundant, with a total of 81 species. Lianas and trees follow, each with about 25 species. Subshrubs, shrubs, and palms are less common. Among the monocots there are 53 species of herbs and 5 species of palms. In contrast, most lianas and trees (25 species) and tree/shrubs (21 species) belong to the eudicots (Figure 23b).

**Figure 24:**
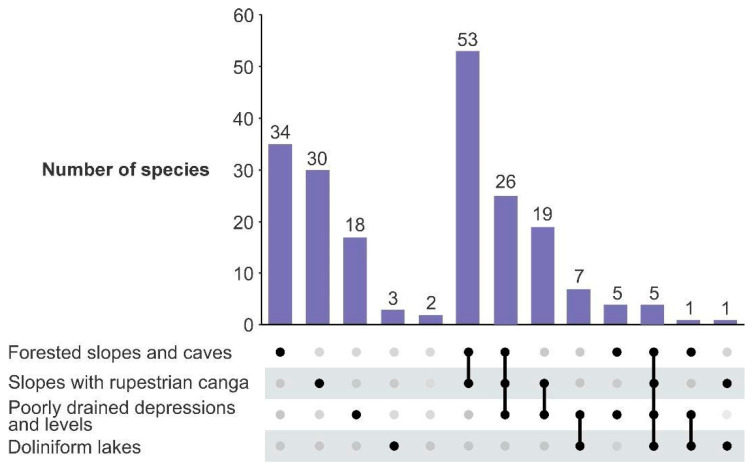
Distribution of species studied according to their occurrences per geoenvironments [12]. 1: slopes with *canga* vegetation over plinthosols; 2: forested slopes and caves over plinthosols and ferralsols; 3: poorly drained depressions and levels over plinthosols and histosols; 4: doliniform lakes with organic mud sediment at bottom; and 5: anthropic areas. This latter is not geoenvrionment, and, thus, it was not counted.

Most of the studied species can be found in forested slopes and caves over plinthosols and ferralsols and slopes with *canga* vegetation over plinthosols (Figure 24). Exclusive occurrences in forested slopes correspond to 34 species, while 30 species are exclusively found in slopes with *canga*. In addition, 18 and 3 species are restricted to poorly drained depressions and levels and doliniform lakes, respectively.

This structural distribution reflects adaptations to the challenging edaphic conditions of the ferruginous environment, where shallow, nutrient-poor soils demand distinct survival strategies across plant groups [12,15]. The presence of diverse life forms also underscores the habitat’s ecological complexity and resilience and the integrity of plant–pollinator interactions [50]. Unfortunately, these essential relationships are increasingly at risk due to widespread human activities [13,37].

A recent study of pre-Columbian influences on Amazonian forests found more cultivated plant species in forests near archaeological sites [47]. This shows that people practiced plant domestication in these areas for a long time. The research resulted in a list of 51 plant species that are in the early stages of domestication, providing strong evidence of their cultivation and management over time. Additionally, useful plants found near the archeological sites of Serra de Carajás were compared to our palynological database to offer an integrated perspective on potential domesticated plants used by both ancient and contemporary Indigenous people. As a result, 17 species (3 palms and 14 eudicots) have been identified with multiple uses, including food supply, building materials, various medicinal applications, firewood, and hunting strategies (Table S1).

## 5. Conclusions

This pollen atlas has important implications for future research. It serves as a reference framework for palynological studies, assisting in the reconstruction of the vegetation, the analysis of the climate history and pre-Columbian influences on vegetation patterns, and the monitoring of ecological changes. Additionally, these findings improve our understanding of plant–pollinator interactions, which are essential for conserving biodiversity in ironstone outcrops that are increasingly threatened by changes in land use and land cover. Future studies could investigate temporal changes in pollen diversity to evaluate the impacts of environmental disturbances, helping to ensure the preservation of this ecologically unique region.

## Figures and Tables

**Table 1:** Pollen and spore morphology of Amazon *canga* vegetation. Shape classes follow [40].

Species	Symmetry	Shape	Aperture Type	Ornamentation	Equatorial Diameter (E)	Polar Diameter (P)	Diameter (D)
Ferns and Lycophytes							
Family: Aspleniaceae Newman							
*Asplenium serratum* L.	bilateral	elliptical	monolete	laevigate to scabrate	50.6–56 µm	29–39 µm	
Family: Blechnaceae Newman							
*Blechnum polypodioides* Raddi	bilateral	elliptical	monolete	laevigate to microreticulate	36–47.6 µm	28–34 µm	
Family: Dennstaedtiaceae Lotsyaz							
*Pteridium esculentum* (G. Forst.) Cockayne	heteropolar	elliptical	monolete	densely rugulate	34–41 µm	30.4–35 µm	
Family*: Isoëtaceae* Rchb.f.							
*Isoëtes cangae J.B.S.Pereira*, *Salino and Stützel*	heteropolar	elliptical	monolete	microechinate	30–34 µm	23–29 µm	
*Isoëtes serracarajensis* J.B.S.Pereira, Salino and Stützel	heteropolar	elliptical	monolete	laevigate and microechinate to tuberculate	30–37 µm	25–30 µm	
Family: Pteridaceae E.D.M.Kirchn.							
*Hemionitis palmata* L.	radial	triangular–obtuse–convex	trilete	densely echinate	27–31 µm	30–35.4 µm	
*Pteris denticulata* Sw.	radial	Triangular–obtuse–straight		densely hamulate	28–35 µm	45–53 µm	
*Pteris pungens* Willd.	radial	Triangular–obtuse–straight		verrucate to hamulate	50–53 µm	40–47 µm	
Family: Thelypteridaceae Pic.Serm.							
*Christella hispidula* (Decne.) Holttum	bilateral	elliptical	monolete	laevigate to hamulate	35–39.2 µm	24–26.2 µm	
*Cyclosorus interruptus* (Willd.) H. Ito	bilateral	elliptical	monolete	densely hamulate–baculate	54–65 µm	36–37.3 µm	
Monocots							
Family: Araceae Juss.							
*Philodendron wullschlaegelii* Schott	heteropolar	oblate	monocolpate	striate	48–57 µm	37–41.5 µm	
Family: Arecaceae Bercht. and J.Presl.							
*Acrocomia aculeata* (Jacq.) Lodd. ex Mart.	heteropolar	oblate spheroidal	trichotomosulcate	microreticulate	83–92 µm	79–89 µm	
*Attalea maripa* (Aubl.) Mart	heteropolar	prolate to perprolate	monosulcate	microreticulate	26.7–30.8 µm	44–51 µm	
*Euterpe oleraceae* Mart.	heteropolar	perprolate	monosulcate	microreticulate	24–27 µm	55–60 µm	
*Oenocarpus distichus* Mart.	heteropolar	perprolate	trichotomosulcate	microreticulate	24–31 µm	47–55 µm	
*Socratea exorrhiza* (Mart.) H.Wendl.	apolar	prolate	monosulcate	scabrate to echinate			52–60 µm
Family: Bromeliaceae Juss.							
*Aechmea bromeliifolia* (Rudge) Baker	heteropolar	oblate spheroidal	di(tri)porate	reticulate to rugulate	65 µm	63.5 µm	
*Aechmea castelnavii* Baker	apolar	spheroidal	inaperturate	heterobrochate reticulate			27–34 µm
*Aechmea mertensii* (G.Mey.) Schult. and Schult.f.	apolar	spheroidal	inaperturate	heterobrochate reticulate			55–50 µm
*Dyckia duckei* L.B.Sm.	heteropolar	subprolate to prolate	monosulcate	heterobrochate reticulate	19–26 µm	41–46 µm	
*Pitcairnia lanuginosa* Ruiz and Pav.	heteropolar	prolate spheroidal	monosulcate	heterobrochate reticulate	41.3–48 µm	49–52 µm	
Family: Costaceae Nakai							
*Chamaecostus acualis* (S.Moore) T.André and C.D.Specht	isopolar	spheroidal	pantoporate	psilate to punctate			139–142 µm
*Chamaecostus lanceolatus* (Petersen) C.D.Specht and D.W.Stey	isopolar	spheroidal	pantoporate	psilate to punctate			119–125 µm
*Costus scaber* Ruiz and Pav.	isopolar	spheroidal	pantoporate	psilate to punctate			204–212 µm
Family: Cyperaceae Juss.							
*Bulbostylis paraensis C.B.Clarke*	heteropolar	spheroidal	inaperturate	microreticulate			39–42 µm
*Cyperus aggregatus* (Willd.) Endl.	heteropolar	spheroidal	inaperturate	microreticulate			31–33 µm
*Cyperus amabilis* Vahl	heteropolar	spheroidal	inaperturate	microreticulate			30–34 µm
*Cyperus haspan* L.	heteropolar	elliptical	inaperturate	microreticulate			25–29 µm
*Cyperus laxus* Lam.	heteropolar	elliptical	inaperturate	microreticulate			29–23 µm
*Cyperus sphacelatus* Rottb.	heteropolar	elliptical	inaperturate	microreticulate			16–21 µm
*Cyperus surinamensis* Rottb.	heteropolar	elliptical	inaperturate	microreticulate			18–21 µm
*Eleocharis flavescens* (Poir.) Urb.	heteropolar	elliptical	inaperturate	microreticulate			21–27 µm
*Rhynchospora barbata* (Vahl) Kunth	heteropolar	elliptical	inaperturate	microreticulate			25–30 µm
*Rhynchospora corymbosa* (L.) Britton	heteropolar	elliptical	inaperturate	microreticulate			17–23 µm
*Rhynchospora seccoi* C.S. Nunes, P.J.S. Silva Filho, and A. Gil	heteropolar	spheroidal	inaperturate	microreticulate			27–32 µm
*Rhynchospora tenuis* Link	heteropolar	spheroidal	inaperturate	microreticulate			22–29 µm
*Scleria cyperina* Willd. ex Kunth	heteropolar	spheroidal	inaperturate	microreticulate			34–38 µm
*Scleria verticillata* Muhl. ex Willd	heteropolar	spheroidal	inaperturate	microreticulate			31–33 µm
Family: *Dioscoreaceae* (*R. Br.*)							
*Dioscorea glandulosa* (Klotzsch ex Griseb.) Kunth	apolar	spheroidal	inaperturate	microreticulate			29–31 µm
*Dioscorea pohlii* Griseb.		elliptical	inaperturate	striate			38–52 µm
*Family*: Eriocaulaceae Martinov							
*Eriocaulon aff. setaceum* L.	apolar	spheroidal	spiraperturate	microechinate and microreticulate			42–47 µm
*Eriocaulon setaceum* L.	apolar	spheroidal	spiraperturate	microechinate and microreticulate			22–25 µm
*Paepalanthus aff. fasciculatus* (Rottb.) Kunth	apolar	spheroidal	spiraperturate	microechinate and microreticulate			41–44 µm
*Syngonanthus caulescens* (Poir.) Ruhland	apolar	spheroidal	spiraperturate	microechinate and microreticulate			26–31 µm
*Syngonanthus discretifolius* (Moldenke) M.T.C.Watan.	apolar	spheroidal	spiraperturate	microechinate and microreticulate			29–34 µm
*Syngonanthus heteropeplus* (Koern.) Ruhland	apolar	spheroidal	spiraperturate	microechinate and microreticulate			26–34 µm
*Syngonanthus* sp. 1	apolar	spheroidal	spiraperturate	microechinate and microreticulate			26–30 µm
Family: Heliconiaceae Nakai							
*Heliconia adeliana* Emygdio and E.Santos	apolar	spheroidal	inaperturate	punctate			70–73 µm
Family: Mayacaceae *Kunth*							
*Mayaca fluviatilis* Aubl.	heteropolar	elliptical	inaperturate	microreticulate			20–31 µm
Family: Poaceae Barnhart							
*Axonopus capillaris* (Lam.) Chase	apolar	spheroidal	monoporate	microreticulate–microrugulate			27–26 µm
*Axonopus carajasensis* Bastos	apolar	spheroidal	monoporate	microreticulate			31–34 µm
*Axonopus longispicus* (Döll) Kuhlm	apolar	spheroidal	monoporate	microreticulate			30–36 µm
*Eragrostis maypurensis* (Kunth) Steud	apolar	spheroidal	monoporate	microreticulate			20–31 µm
*Eragrostis rufescens* Schrad. ex Schult	apolar	spheroidal	monoporate	microreticulate			32–39 µm
*Hildaea breviscrobs* (Döll) C.Silva and R.P. Oliveira	apolar	spheroidal	monoporate	microreticulate			20–23 µm
*Ichnanthus calvescens* (*Nees ex Trin.*) *Döll*	apolar	spheroidal	monoporate	microreticulate			34–42 µm
*Isachne polygonoides* (Lam.) Döll	apolar	spheroidal	monoporate	microreticulate			21–28 µm
*Mesosetum cayennense* Steud.	apolar	spheroidal	monoporate	microreticulate			32–41 µm
*Mnesithea aurita* (Steud.) de Koning and Sosef	apolar	spheroidal	monoporate	microreticulate			32–36 µm
*Otachyrium versicolor* (Döll) Henrard	apolar	spheroidal	monoporate	microreticulate			31–41 µm
*Paspalum carajasense* S.Denham	apolar	spheroidal	monoporate	microreticulate			51–61 µm
*Paspalum carinatum* Humb. and Bonpl. ex Flüggé	apolar	spheroidal	monoporate	microreticulate			42–63 µm
*Paspalum virgatum* L.	apolar	spheroidal	monoporate	microreticulate			42–58 µm
*Rhytachne gonzalezii* Davidse	apolar	spheroidal	monoporate	microreticulate			48–65 µm
*Sporobolus multiramosus* Longhi-Wagner and Boechat	apolar	spheroidal	monoporate	microreticulate–reticulate			31–35 µm
*Trichanthecium parvifolium* (Lam.) Zuloaga and Morrone	apolar	spheroidal	monoporate	microreticulate–reticulate			34–40 µm
*Trichanthecium polycomum* (Trin.) Zuloaga and Morrone	apolar	spheroidal	monoporate	microreticulate–reticulate			29–37 µm
*Trichanthecium* sp. 1	apolar	spheroidal	monoporate	microreticulate			29–32 µm
Family: Velloziaceae *J.Agardh*							
*Vellozia* sp. 1	apolar	spheroidal	inaperturate	reticulate			80–85 µm
*Vellozia* sp. 2	apolar	spheroidal	inaperturate	reticulate			62–70 µm
Family: Xyridaceae *C.Agardh*							
*Xyris brachysepala* Kral.	heteropolar	elliptical	inaperturate	reticulate			34–62 µm
*Xyris macrocephala* Vahl	heteropolar	elliptical	inaperturate	microreticulate			35–48 µm
EUDICOTS AND MAGNOLIIDS							
Family: Acanthaceae Juss.							
*Justicia birae* A.S.Reis, F.A.Silva, A.Gil, and Kameyama	isopolar	prolate	dicolporate	echinate and microreticulate	58–60 µm.	98–105 µm	
Family: Anacardiaceae R.Br.							
*Anacardium occidentale* L.	isopolar	prolate/oblate spheroidal	tricolporate	striate–reticulate	44–55 µm	50–59 µm	
Family: Annonaceae Juss.							
*Onychopetalum amazonicum* R.E.Fr.	isopolar	subprolate	zonasulcate	rugulate	52.8–57.6 µm	42.8–49 µm	
*Xylopia aromatica* (Lam.) Mart.	isopolar	prolate spheroidal	tricolporate	reticulate	27–29 µm	28–30 µm	
Family: Apocynaceae Juss.							
*Mandevilla hirsuta* (A.Rich.) K.Schum.	isopolar	prolate spheroidal	pantoporate	psilate and microreticulate			100–110 µm
*Mandevilla tenuifolia* (J.C.Mikan) Woodson	isopolar	prolate spheroidal	pantoporate	reticulate			34–48 µm
Family: Asteraceae Bercht. and J.Presl							
*Cavalcantia glomerata* (G.M.Barroso and R.M.King) R.M.King, and H.Rob.	isopolar	prolate spheroidal	tricolporate	echinate and punctate	20–25 µm	22–25 µm	
*Emilia* sp. 1	isopolar	prolate spheroidal	tricolporate	echinate and punctate	23–24 µm	24–25 µm	
*Ichthyothere terminalis* (Spreng.) S.F.Blake	isopolar	prolate spheroidal	tricolporate	echinate and punctate	32–33 µm	31–38 µm	
*Lepidaploa paraensis* (H.Rob.) H.Rob	isopolar	oblate spheroidal	tricolporate	echinate	40–55 µm	40–54 µm	
*Monogereion carajensis* G.M.Barroso and R.M.King	isopolar	oblate spheroidal	tricolporate	echinate	20–27 µm	20–28 µm	
*Riencourtia pedunculosa* (Rich.) Pruski	isopolar	subprolate	tricolporate	echinate	28–30 µm	31–33 µm	
Family: Begoniaceae C.Agardh							
*Begonia guaduensis* Kunth	isopolar	prolate	tricolporate	psilate and slightly striate	11–12 µm	20–24.4 µm	
Family: Bignoniaceae Juss.							
*Anemopaegma carajasense* A.H. Gentry ex Firetti-Leggieri	isopolar	circular	stephanocolpate	heterobrochate reticulate			110–124 µm
*Handroanthus serratifolius* (Vahl) S.Grose	isopolar	subprolate	tricolporate	homobrochate reticulate	52–70 µm	70–80 µm	
*Jacaranda copaia* (Aubl.) D.Don	isopolar	prolate	tricolporate	microreticulate	33–55 µm	50–58 µm	
Family: Bixaceae Kunth							
*Bixa orellana* L.	isopolar	subprolate	tricolporate	microreticulate	41–63 µm	54–65 µm	
Family: Cactaceae Juss.							
*Cereus hexagonus* (L.) Mill.	isopolar	oblate spheroidal	tricolpate	reticulate, echinate e microequinate	110–115 µm	98–105 µm	
Family: Clusiaceae Lindl.							
*Clusia nemorosa* G.Mey.	isopolar	prolate spheroidal	tricolporate	microreticulate	29.4–38 µm	34.3–42.4 µm	
Family: Convolvulaceae Juss.							
*Aniseia cernua* Moric.	apolar	spheroidal	pantocolpate	microreticulate and microequinate			76–83 µm
*Cuscuta insquamata* Yunck.	isopolar	subprolate	tricolpate	microreticulate and microequinate	18–39 µm	25–48 µm	
*Distimake macrocalyx* (Ruiz and Pav.) A.R. Simões and Staples	isopolar	subprolate	tricolpate	microreticulate and microgranulate	72–83 µm	69–86.6 µm	
*Evolvulus filipes* Mart.	apolar	spheroidal	pantocolpate	microreticulate			28–32 µm
*Evolvulus* sp. 1	isopolar	prolate spheroidal	pantocolpate	microreticulate	69–92 µm	71–93 µm	
*Ipomoea asplundii* O’Donell	apolar	spheroidal	pantoporate	echinate and microreticulate			78–82 µm
*Ipomoea carajasensis* D.F. Austin	apolar	spheroidal	pantoporate	echinate and microreticulate			80–90 µm
*Ipomoea cavalcantei* D.F. Austin	apolar	spheroidal	pantoporate	echinate and microreticulate			84–155 µm
*Ipomoea cavalcantei x marabaensis*	apolar	spheroidal	pantoporate	echinate and microreticulate			85–87 µm
*Ipomoea decora* Meisn.	apolar	spheroidal	pantoporate	echinate and microreticulate			94–103 µm
*Ipomoea goyazensis* Gardner	apolar	spheroidal	pantoporate	echinate and microreticulate			118–125 µm
*Ipomoea marabaensis* D.F.Austin and Secco	apolar	spheroidal	pantoporate	echinate and microreticulate			120–145 µm
*Ipomoea procumbens* Mart. ex Choisy	apolar	spheroidal	pantoporate	echinate and microreticulate			113–117 µm
*Ipomoea setifera* Poir.	apolar	spheroidal	pantoporate	echinate and microreticulate			98–102 µm
*Jacquemontia tamnifolia* (L.) Griseb.	apolar	spheroidal	pantocolporate	microechinate and microreticulate			60–65 µm
*Turbina cordata* (Choisy) D.F.Austin and Staples	apolar	spheroidal	pantoporate	echinate and microreticulate			156–160 µm
Family: Erythroxylaceae Kunth							
*Erythroxylum carajasense* Plowman	isopolar	prolate spheroidal	tricolporate	heterobrochate reticulate	29 µm	33 µm	
*Erythroxylum nelson-rosae* Plowman	isopolar	prolate spheroidal	tricolporate	heterobrochate reticulate	45–52 µm	56–60 µm	
Family: Euphorbiaceae Juss.							
*Alchornea discolor* Poepp.	isopolar	prolate spheroidal to subprolate	tricolporate	microreticulate	24–25 µm	26–28 µm	
*Aparisthmium cordatum* (A.Juss.) Baill.	isopolar	subspheroidal	tricolporate	rugulate–punctate	32–39 µm	25–30 µm	
*Astraea lobata* (L.) Klotzsch	isopolar	spheroidal	inaperturate	croton pattern			59–63 µm
*Sapium glandulosum* (L.) Morong	isopolar	prolate	tricolporate	reticulate	40–45 µm	60–68 µm	
Family: Fabaceae Juss.							
*Abrus fruticulosus* Wight and Arn.	isopolar	prolate	tricolporate	psilate to punctate	39–45.8 µm	31–32 µm	
*Aeschynomene rudis* Benth.	isopolar	subprolate	tricolporate	heterobrochate microreticulate	20.1–22 µm	23.5–29 µm	
*Andira inermis* (W.Wright) DC.	isopolar	prolate spheroidal	tricolporate	microreticulate	20–22.6 µm	21–26.3 µm	
*Bauhinia pulchella* Benth.	isopolar	oblate	tricolporate	microreticulate	130–165 µm	60–70 µm	
*Centrosema carajasense* Cavalcante	isopolar	oblate	stephanocolpate	microreticulate	56–59 µm	28–33.2 µm	
*Cerradicola elliptica* (Desv) L.P.Queiroz	isopolar	subprolate	tricolporate	heterobrochate reticulate	43–44 µm	53–54 µm	
*Chamaecrista desvauxii* (Collad.) Killip	isopolar	perprolate	tricolporate	psilate	34–41.3 µm	69.1–79.4 µm	
*Chamaecrista flexuosa* (L.) Greene	isopolar	prolate	tricolporate	microreticulate	50 µm	84–85 µm	
*Chamaecrista* sp. 1	isopolar	prolate	tricolporate	microreticulate	45.7–56.6 µm	80–90 µm	
*Clitoria fairchildiana* R.A.Howard	isopolar	oblate	pantocolpate	microreticulate and punctate	72–77 µm	39–45 µm	
*Copaifera martii* Hayne	isopolar	subprolate	tricolporate	psilate and scabrate	30–40 µm	20–30 µm	
*Crotalaria maypurensis* Kunth	isopolar	prolate	tricolporate	microreticulate	22–28 µm	36–45 µm	
*Dioclea apurensis* Kunth	isopolar	oblate	parasyncolporate	rugulate to reticulate	75–80 µm	40 µm	
*Dioclea virgata* (Rich.) Amshoff	isopolar	peroblate	tricolporate	reticulate	82.3–94.8 µm	39.5–47 µm	
*Dipteryx odorata* (Aubl.) Forsyth F.	isopolar	subspheroidal	parasyncolporate	microreticulate	40–54 µm	46–60 µm	
*Mimosa acutistipula* var. *ferrea* Barneby	apolar	elliptical	tetrad calymmate	microreticulate and psilate			10–14 µm
*Mimosa aff. skinneri* Benth.	apolar	spheroidal	tetrad calymmate	psilate			7–9 µm
*Mimosa carajarum* (Barneby) T.P.Mendes and M.J.Silva	apolar	spheroidal	tetrad calymmate	psilate			7–8 µm
*Mimosa somnians var. viscida* (Willd.) Barneby	apolar	elliptical	tetrad calymmate	microreticulate			13–20 µm
*Mimosa xanthocentra* Mart.	apolar	elliptical	tetrad calymmate	microreticulate			18–20 µm
*Mimosa xanthocentra* var. *mansii* (Mart.) Barneby	apolar	spheroidal	tetrad calymmate	psilate			7–8 µm
*Parkia platycephala* Benth.	apolar	elliptical	polyads calymmate	psilate to scabrate			160–185 µm
*Periandra coccinea* (Schrad.) Benth.	isopolar	subprolate	tricolporate	punctate	53–53 µm	62–64 µm	
*Periandra mediterranea* (Vell.) Taub.	isopolar	prolate	tricolporate	microreticulate	34–39 µm	36–44 µm	
*Schizolobium parahyba var. amazonicum* (Huber ex Ducke) Barneby	isopolar	prolate spheroidal	tricolporate	heterobrochate reticulate	29.8–36.1 µm	37–43.5 µm	
*Senna multijuga* (Rich.) H.S.Irwin and Barneby	isopolar	prolate	tricolporate	microreticulate	30–48 µm	50–58 µm	
*Senna siamea* (Lam.) H.S.Irwin and Barneby	apolar	prolate spheroidal	tricolporate	rugulate and reticulate			49–50 µm
*Stryphnodendron pulcherrimum* (Willd.) Hochr.	apolar	elliptical	polyads calymmate	psilate to scabrate			25–32 µm
*Stylosanthes humilis* Kunth	isopolar	prolate	tricolporate	microreticulate	41–50.7 µm	62–69 µm	
*Tachigali vulgaris* L.G.Silva and H.C.Lima	isopolar	prolate	tricolporate	microreticulate	22–25 µm	30–35 µm	
Family: Gentianaceae Juss.							
*Chelonanthus purpurascens* (Aubl.) Struwe et al.	apolar	triangular	3-hemicolporate	reticulate			70–80 µm
*Schultesia benthamiana* Klotzsch ex Griseb	apolar	triangular	3-hemicolporate	reticulate			80–92 µm
Family: Hypericaceae Juss.							
*Vismia cayennensis* (Jacq.) Pers.	isopolar	prolate	parasyncolporate	heterobrochate reticulate	30–38 µm	40–52 µm	
Family: Lamiaceae Lindl.							
*Hyptis atrorubens* Poit.	isopolar	oblate	stephanocolpate	heterobrochate reticulate	30–40 µm	15–20 µm	
*Hyptis parkeri* Benth.	isopolar	oblate	stephanocolpate	heterobrochate reticulate	35–45 µm	20–25 µm	
Family: Lecythidaceae A.Juss.							
*Bertholletia excelsa* Bonpl.	isopolar	subprolate	tricolporate	microreticulate	29–32.8 µm	42–50 µm	
Family: Lentibulariaceae Rich							
*Utricularia pusilla* Vahl	isopolar	subprolate	stephanocolporate	psilate to scabrate	15–17 µm	18–23 µm	
*Utricularia* sp. 1	isopolar	subprolate	stephanocolporate	psilate to scabrate	10.9–13.4 µm	15–17.5 µm	
*Utricularia* sp. 2	isopolar	prolate spheroidal	stephanocolporate	microreticulate	23–27 µm	26–30 µm	
Family: Lythraceae J.St.-Hil.							
*Cuphea annulata* Koehne	isopolar	oblate	syncolporate	striate	33.8–37.2 µm	22–17.4 µm	
*Cuphea carajasensis* Lourteig	isopolar	oblate	syncolporate	striate	26–29 µm	16–20 µm	
*Cuphea* sp. 1	isopolar	oblate	syncolporate	striate	33–37.6 µm	23.3–29.9 µm	
*Cuphea* sp. 2	isopolar	subprolate	parasyncolporate	psilate	17–21 µm	22–24 µm	
*Cuphea* sp. 3	isopolar	oblate	tricolporate	microreticulate	21–24.7 µm	18–21 µm	
Family: Malpighiaceae Juss.							
*Banisteriopsis appressa* (B.Gates) R.F.Almeida and M.Pell.	apolar	quadrangular	pantoporate	microreticulate			43–54 µm
*Banisteriopsis malifolia* (Nees and Mart.) B.Gates	apolar	spheroidal to quadrangular	pantoporate	microreticulate			50–55 µm
*Banisteriopsis* sp. 1	apolar	spheroidal to hexangular	pantoporate	microreticulate			60–70 µm
*Byrsonima chrysophylla* Kunth	isopolar	spheroidal	tricolporate	microreticulate	19–21 µm	20–23 µm	
*Byrsonima spicata* (Cav.) DC.	isopolar	spheroidal	tricolporate	microreticulate	14.2–14.9 µm	15.8–14.2 µm	
*Diplopterys pubipetala* (A.Juss.) W.R.Anderson and C.C.Davis	apolar	spheroidal to quadrangular	pantoporate	microreticulate			50–60 µm
*Spachea lactescens* (Ducke) R.F.Almeida and M.Pell.	isopolar	prolate spheroidal	tricolporate	microreticulate	22–25 µm	22–26 µm	
Family: Malvaceae Juss.							
*Guazuma ulmifolia* Lam.	isopolar	prolate spheroidal	tricolporate	heterobrochate reticulate	22–23 µm	22–24 µm	
*Melochia arenosa* Benth.	isopolar	prolate spheroidal	tricolporate	microreticulate	53–54.7 μm	54.4–57 μm	
*Melochia spicata* (L.) Fryxell	isopolar	oblate spheroidal	tricolporate	microreticulate	45–52 μm	45–48 μm	
*Theobroma grandiflorum* (Willd. ex Spreng.) K.Schum.	isopolar	prolate spheroidal	tricolporate	heterobrochate reticulate	22–30 μm	24–32 μm	
Family: Marcgraviaceae Bercht. and J.Presl							
*Norantea guianensis* Aubl.	isopolar	oblate spheroidal to prolate	tricolporate	microreticulate to scabrate	27–37 μm	26–36 μm	
Family: Melastomataceae Juss.							
*Miconia chamissois* Naudin	isopolar	subprolate to prolate	tricolporate	psilate	28–32 µm	36–40 µm	
*Pleroma stenocarpum* (Schrank et Mart. ex DC.) Triana	isopolar	subprolate	tricolporate	psilate	17–20 µm	20–24 µm	
Family: Meliaceae Juss.							
*Carapa guianensis* Aubl.	isopolar	oblate spheroidal	pantocolporate	microreticulate	52–61 µm	50–60 µm	
Family: Menyanthaceae Dumort.							
*Nymphoides humboldtiana* (Kunth) Kuntze	isopolar	oblate	parasyncolporate	microreticulate	42–55 µm	30–35 µm	
Family Myristicaceae R.Br.							
*Virola michelii* Heckel	heteropolar	oblate to suboblate	monocolpate	heterobrochate reticulate	30–44 µm	25–28 µm	
Family: Myrtaceae Juss.							
*Eugenia flavescens* DC.	isopolar	oblate	syncolporate	microreticulate	18–22 µm	12–14 µm	
*Eugenia punicifolia* (Kunth) DC.	isopolar	oblate	parasyncolporate	microreticulate to scabrate	24–28 µm	15–18 µm	
*Myrcia multiflora* (Lam.) DC.	isopolar	oblate	syncolporate	microreticulate to scabrate	20–25 µm	15–18 µm	
Family: Passifloraceae Juss. ex Roussel							
*Passiflora glandulosa Cav.*	isopolar	prolate spheroidal	3-mesocolpi fused in pairs	reticulate	88–92 µm	90–94 µm	
*Passiflora tholozanii* Sacco	isopolar	prolate spheroidal	3-mesocolpi fused in pairs	reticulate	68–80 µm	70–82 µm	
Family: Phyllanthaceae Martinov							
*Phyllanthus hyssopifolioides* Kunth	isopolar	perprolate	tricolporate	heterobrochate reticulate	15–17 µm	38–42 µm	
Family: Polygalaceae Hoffmanns. and Link							
*Caamembeca spectabilis* (DC.) J.F.B.Pastore	isopolar	prolate	stephanocolporate	microreticulate to perforate	69–70 µm	100–110 µm	
*Securidaca diversifolia* (L.) S.F.Blake	isopolar	prolate	stephanocolporate	microreticulate to perforate	30–35 µm	40–46 µm	
*Senega adenophora* (DC.) J.F.B.Pastore	isopolar	prolate	stephanocolporate	microreticulate	75–80 µm	40–46 µm	
Family: Rubiaceae Juss.							
*Borreria alata* (Aubl.) DC.	apolar	spheroidal	pantoporate	puntate with microechinae			35–40 µm
*Borreria elaiosulcata* E.L.Cabral and L.M.Miguel	apolar	spheroidal	pantoporate	puntate with microechinae			33–40 µm
*Borreria latifolia* (Aubl.) K.Schum	apolar	spheroidal	pantoporate	puntate with microechinae			50–60 µm
*Borreria paraensis* E.L.Cabral and Bacigalupo	apolar	spheroidal	pantoporate	puntate with microechinae			20–24 µm
*Carajasia cangae* R.M.Salas, E.L.Cabral and Dessein	apolar	spheroidal	stephanocolporate	microreticulate			25–30 µm
*Ixora coccinea* L.	isopolar	prolate spheroidal	tricolporate	heterobrochate reticulate	35–40 µm	35–40 µm	
*Mitracarpus carajasensis* E.L.Cabral, Sobrado, and E.B.Souza	isopolar	prolate spheroidal	pantocolpate	puntate with microechinae	20–24 µm	20–24 µm	
*Perama carajensis* J.H.Kirkbr.	isopolar	prolate spheroidal	pantocolporate	microreticulate	44–50 µm	44–50 µm	
*Perama* sp. 1	isopolar	prolate spheroidal	tricolporate	rugulate	30–33 µm	30–33 µm	
*Spermacoce* sp. 1	apolar	spheroidal	pantoporate	microreticulate			30–35 µm
*Spermacoce* sp. 2	apolar	spheroidal	pantoporate	microreticulate			27–30 µm
Family: Rutaceae Juss.							
*Pilocarpus microphyllus* Stapf ex Wardlew.	isopolar	prolate	tricolporate	heterobrochate reticulate	20–26 µm	25–34 µm	
*Zanthoxylum gardneri* Engl.	isopolar	prolate spheroidal	tricolporate	heterobrochate reticulate	16–17 µm	20–24 µm	
Family: Sapindaceae Juss.							
*Serjania caracasana* (Jacq.) Willd.	heteropolar	oblate	syncolporate	microreticulate	47–53 µm	35–38 µm	
Family: Solanaceae Adans.							
*Solanum crinitum* Lam.	isopolar	prolate	tricolporate	microreticulate	32–34 µm	32–35 µm	
Family: Styracaceae DC. and Spreng.							
*Styrax ferrugineus* Nees and Mart	isopolar	prolate spheroidal	tricolporate	microreticulate	46–54 µm	48–56 µm	
Family: Turneraceae Kunth ex DC.							
*Turnera glaziovii* Urb.	isopolar	prolate	dicolporate	microreticulate	55–58 µm	105–110 µm	
Family: Verbenaceae J.St.-Hil.							
*Lantana* sp. 1	isopolar	spheroidal	stephanocolpate	heterobrochate reticulate	38–42 µm	38–42 µm	
*Lippia grata* Schauer	isopolar	prolate spheroidal	tricolporate	microreticulate	25–30 µm	30–34 µm	
Family: Vitaceae Juss.							
*Cissus erosa* Rich.	isopolar	prolate	tricolporate	microreticulate	35–44 µm	55–64 µm	
Family: Vochysiaceae A.St.-Hil.							
*Callisthene microphylla* Warm.	isopolar	prolate	tricolporate	psilate	18–20	18–20 µm	

## Data Availability

The ecological data presented in this article can be found in the Reflora database (https://reflora.jbrj.gov.br/reflora/herbarioVirtual/, accessed on 23 September 2024), which represents a virtual herbarium.

## Supplementary Materials

The following supporting information can be downloaded at https://www.mdpi.com/article/10.3390/plants14091319/s1: Table S1. Summary of the plant species from the canga vegetation with pollen and spores described in the palynological atlas with pollination syndromes according to Giannini et al. (2021) [46], degree of domestication (Levis et al., 2017) [47] and main uses (Santos et al., 2019) [48]. NA: not available. Data S1. Pollen key of the Palynological Atlas of Amazon canga vegetation.
