# Supplementary material for: A Palynological Atlas of the Amazon canga Vegetation
Source: Plants (Basel). 2025 Apr 27;14(9):1319. doi: 10.3390/plants14091319 (PMC12073343; doi:10.3390/plants14091319)
Supplement: Supplementary file 1 [file plants-14-01319-s001.zip › Data S1- Pollen Key (md TG21ABR25).pdf]

Data S1. Pollen key of the Palynological Atlas of Amazon *canga* vegetation

1. Pollen grains calymmate

1.1. Aperture: polyads calymmate

1.1.1. Shape: elliptical

1.1.1.1. Ornamentation: psilate to scabrate

1.1.1.1.1. Size medium → *Stryphnodendron pulcherrimum*

1.1.1.1.2. Size very large → *Parkia platycephala*

1.2. Aperture: tetrad calymmate

1.2.1. Shape: elliptical

1.2.1.1. Ornamentation: microreticulate

1.2.1.1.1. Size small → *Mimosa somnians*

Mimosa xanthocentra

1.2.1.2. Ornamentation: microreticulate and psilate

1.2.1.2.1. Size small → *Mimosa acutistipula*

1.2.2. Shape: spheroidal

1.2.2.1. Ornamentation: psilate

1.2.2.1.1. Size very small → *Mimosa aff.*

Mimosa carajarum

Mimosa xanthocentra

2. Pollen grains colpate

2.1. Aperture: monocolpate

2.1.1. Shape: oblate

2.1.1.1. Ornamentation: striate

2.1.1.1.1. Size large → *Philodendron wullschlaegelii*

2.1.2. Shape: oblate to suboblate

2.1.2.1. Ornamentation: reticulate heterobrochate

2.1.2.1.1. Size medium → *Virola michelii*

2.2. Aperture: pantocolpate

2.2.1. Shape: prolate spheroidal

2.2.1.1. Ornamentation: microreticulate

2.2.1.1.1. Size large → *Evolvulus sp.*

2.2.1.2. Ornamentation: puntate with microechinae

2.2.1.2.1. Size small → *Mitracarpus carajasensis*

2.2.2. Shape: spheroidal

2.2.2.1. Ornamentation: microreticulate

2.2.2.1.1. Size medium → *Evolvulus filipes*

2.2.2.2. Ornamentation: microreticulate and microequinate

2.2.2.2.1. Size large → *Aniseia cernua*

2.3. Aperture: stephanocolpate

2.3.1. Shape: circular

2.3.1.1. Ornamentation: reticulate heterobrochate

2.3.1.1.1. Size very large → *Anemopaegma carajasense*

2.3.2. Shape: oblate

2.3.2.1. Ornamentation: microreticulate

2.3.2.1.1. Size large → *Centrosema carajasense*

2.3.2.2. Ornamentation: reticulate heterobrochate

2.3.2.2.1. Size medium → *Hyptis atrorubens*

Hyptis parkeri

2.3.3. Shape: spheroidal

2.3.3.1. Ornamentation: reticulate heterobrochate

2.3.3.1.1. Size medium → *Lantana sp.*

2.4. Aperture: tricolpate

2.4.1. Shape: oblate spheroidal

2.4.1.1. Ornamentation: reticulate, echinate e microequinate

2.4.1.1.1. Size very large → *Cereus hexagonus*

2.4.2. Shape: subprolate

2.4.2.1. Ornamentation: microreticulate and microequinate

2.4.2.1.1. Size medium → *Cuscuta insquamata*

2.4.2.2. Ornamentation: microreticulate and microgranulate

2.4.2.2.1. Size large → *Distimake macrocalyx*

3. Pollen grains colporate

3.1. Aperture: 3-hemicolporate

3.1.1. Shape: triangular

3.1.1.1. Ornamentation: reticulate

3.1.1.1.1. Size large → *Chelonanthus purpurascens*

Schultesia benthamiana

3.2. Aperture: dicolporate

3.2.1. Shape: prolate

3.2.1.1. Ornamentation: echinate and microreticulate

3.2.1.1.1. Size large → *Justicia birae*

3.2.1.2. Ornamentation: microreticulate

3.2.1.2.1. Size very large → *Turnera glaziovii*

3.3. Aperture: pantocolpate

3.3.1. Shape: oblate

3.3.1.1. Ornamentation: microreticulate and punctate

3.3.1.1.1. Size large → *Clitoria fairchildiana*

3.4. Aperture: pantocolporate

3.4.1. Shape: oblate spheroidal

3.4.1.1. Ornamentation: microreticulate

3.4.1.1.1. Size large → *Carapa guianensis*

3.4.2. Shape: prolate spheroidal

3.4.2.1. Ornamentation: microreticulate

3.4.2.1.1. Size medium → *Perama carajensis*

3.4.3. Shape: spheroidal

3.4.3.1. Ornamentation: microechinate and microreticulate

3.4.3.1.1. Size large → *Jacquemontia tamnifolia*

3.5. Aperture: parasyncolporate

3.5.1. Shape: oblate

3.5.1.1. Ornamentation: microreticulate

3.5.1.1.1. Size small → *Nymphoides humboldtiana*

3.5.1.2. Ornamentation: microreticulate to scabrate

3.5.1.2.1. Size small to medium → *Eugenia punicifolia*

3.5.1.3. Ornamentation: rugulate to reticulate

3.5.1.3.1. Size large → *Dioclea apurensis*

3.5.2. Shape: prolate

3.5.2.1. Ornamentation: reticulate heterobrochate

3.5.2.1.1. Size medium to large → *Vismia cayennensis*

3.5.3. Shape: subprolate

3.5.3.1. Ornamentation: psilate

3.5.3.1.1. Size small → *Cuphea sp.*

3.5.4. Shape: subspheroidal

3.5.4.1. Ornamentation: microreticulate

3.5.4.1.1. Size large → *Dipteryx odorata*

3.6. Aperture: stephanocolporate

3.6.1. Shape: prolate

3.6.1.1. Ornamentation: microreticulate

3.6.1.1.1. Size large → *Senega adenophora*

3.6.1.2. Ornamentation: microreticulate to perforate

3.6.1.2.1. Size large to very large → *Caamembeca spectabilis*

3.6.1.2.2. Size medium → *Securidaca diversifolia*

3.6.2. Shape: prolate spheroidal

3.6.2.1. Ornamentation: microreticulate

3.6.2.1.1. Size medium → *Utricularia sp.*

3.6.3. Shape: spheroidal

3.6.3.1. Ornamentation: microreticulate

3.6.3.1.1. Size medium → *Carajasia cangae*

3.6.4. Shape: subprolate

3.6.4.1. Ornamentation: psilate to scabrate

3.6.4.1.1. Size small → *Utricularia pusilla*

Utricularia sp.

3.7. Aperture: syncolporate

3.7.1. Shape: oblate

3.7.1.1. Ornamentation: microreticulate

3.7.1.1.1. Size medium → *Serjania caracasana*

3.7.1.1.2. Size small → *Eugenia flavescens*

3.7.1.2. Ornamentation: microreticulate to scabrate

3.7.1.2.1. Size small → *Myrcia multiflora*

3.7.1.3. Ornamentation: striate

3.7.1.3.1. Size medium → *Cuphea annulata*

Cuphea carajasensis

Cuphea sp.

3.8. Aperture: tricolporate

3.8.1. Shape: oblate

3.8.1.1. Ornamentation: microreticulate

3.8.1.1.1. Size medium → *Cuphea sp.*

3.8.1.1.2. Size very large → *Bauhinia pulchella*

3.8.2. Shape: oblate spheroidal

3.8.2.1. Ornamentation: echinate

3.8.2.1.1. Size medium → *Lepidaploa paraensis*

3.8.2.1.2. Size small → *Monogereion carajensis*

3.8.2.2. Ornamentation: microreticulate

3.8.2.2.1. Size medium to large → *Melochia spicata*

3.8.3. Shape: oblate spheroidal to prolate spheroidal

3.8.3.1. Ornamentation: microreticulate to scabrate

3.8.3.1.1. Size medium → *Norantea guianensis*

3.8.4. Shape: peroblate

3.8.4.1. Ornamentation: reticulate

3.8.4.1.1. Size large → *Dioclea virgata*

3.8.5. Shape: perprolate

3.8.5.1. Ornamentation: psilate

3.8.5.1.1. Size large → *Chamaecrista desvauxii*

3.8.5.2. Ornamentation: reticulate heterobrochate

3.8.5.2.1. Size medium → *Phyllanthus hyssopifolioides*

3.8.6. Shape: prolate

3.8.6.1. Ornamentation: microreticulate

3.8.6.1.1. Size large → *Chamaecrista flexuosa*

Chamaecrista sp.

Crotalaria maypurensis

Stylosanthes humilis

Cissus erosa

3.8.6.1.2. Size medium → *Periandra mediterranea*

Senna multijuga

Tachigali vulgaris

Solanum crinitum

3.8.6.1.3. Size medium to large → *Jacaranda copaia*

3.8.6.2. Ornamentation: psilate

3.8.6.2.1. Size small → *Callisthene microphylla*

3.8.6.3. Ornamentation: psilate and slightly striate

3.8.6.3.1. Size small → *Begonia guaduensis*

3.8.6.4. Ornamentation: psilate to punctate

3.8.6.4.1. Size medium → *Abrus fruticulosus*

3.8.6.5. Ornamentation: reticulate

3.8.6.5.1. Size large → *Sapium glandulosum*

3.8.6.6. Ornamentation: reticulate heterobrochate

3.8.6.6.1. Size medium → *Pilocarpus microphyllus*

3.8.7. Shape: prolate spheroidal

3.8.7.1. Ornamentation: echinate and punctate

3.8.7.1.1. Size medium → *Ichthyothere terminalis*

3.8.7.1.2. Size small → *Cavalcantia glomerata*

Emilia sp.

3.8.7.2. Ornamentation: microreticulate

3.8.7.2.1. Size large → *Melochia arenosa*

Styrax ferrugineus

3.8.7.2.2. Size medium → *Clusia nemorosa*

Lippia grata

3.8.7.2.3. Size small → *Andira inermis*

Spachea lactescens

3.8.7.3. Ornamentation: reticulate

3.8.7.3.1. Size medium → *Xylopia aromatica*

3.8.7.4. Ornamentation: reticulate heterobrochate

3.8.7.4.1. Size large → *Erythroxylum nelson-rosae*

3.8.7.4.2. Size medium → *Erythroxylum carajasense*

Schizolobium parahyba

Ixora coccinea

Zanthoxylum gardneri

3.8.7.4.3. Size small → *Guazuma ulmifolia*

3.8.7.4.4. Size small to medium → *Theobroma grandiflorum*

3.8.7.5. Ornamentation: rugulate

3.8.7.5.1. Size medium → *Perama sp.*

3.8.7.6. Ornamentation: rugulate and reticulate

3.8.7.6.1. Size medium → *Senna siamea*

3.8.8. Shape: prolate spheroidal to subprolate

3.8.8.1. Ornamentation: microreticulate

3.8.8.1.1. Size medium → *Alchornea discolor*

3.8.9. Shape: prolate/oblate spheroidal

3.8.9.1. Ornamentation: striate-reticulate

3.8.9.1.1. Size large → *Anacardium occidentale*

3.8.10. Shape: spheroidal

3.8.10.1. Ornamentation: microreticulate

3.8.10.1.1. Size small → *Byrsonima chrysophylla*

3.8.10.1.2. Size small → *Byrsonima spicata*

3.8.11. Shape: subprolate

3.8.11.1. Ornamentation: echinate

3.8.11.1.1. Size medium → *Riencourtia pedunculosa*

3.8.11.2. Ornamentation: microreticulate

3.8.11.2.1. Size large → *Bixa orellana*

3.8.11.2.2. Size medium → *Bertholletia excelsa*

3.8.11.3. Ornamentation: microreticulate heterobrochate

3.8.11.3.1. Size small → *Aeschynomene rudis*

3.8.11.4. Ornamentation: psilate

3.8.11.4.1. Size small → *Pleroma stenocarpum*

3.8.11.5. Ornamentation: psilate and scabrate

3.8.11.5.1. Size medium → *Copaifera martii*

3.8.11.6. Ornamentation: punctate

3.8.11.6.1. Size large → *Periandra coccinea*

3.8.11.7. Ornamentation: reticulate heterobrochate

3.8.11.7.1. Size large → *Cerradicola elliptica*

3.8.11.8. Ornamentation: reticulate homobrochate

3.8.11.8.1. Size large → *Handroanthus serratifolius*

3.8.12. Shape: subprolate to prolate

3.8.12.1. Ornamentation: psilate

3.8.12.1.1. Size medium → *Miconia chamissois*

3.8.13. Shape: subspheroidal

3.8.13.1. Ornamentation: rugulate-punctate

3.8.13.1.1. Size medium → *Aparisthmium cordatum*

4. Pollen grains inaperturate

4.1. Aperture: inaperturate

4.1.1. Shape: elliptical

4.1.1.1. Ornamentation: microreticulate

4.1.1.1.1. Size medium → *Cyperus laxus*

Eleocharis flavescens

Rhynchospora barbata

Rhynchospora corymbosa

Mayaca fluviatilis

Xyris macrocephala

4.1.1.1.2. Size small → *Cyperus sphacelatus*

Cyperus surinamensis

4.1.1.2. Ornamentation: reticulate

4.1.1.2.1. Size large → *Xyris brachysepala*

4.1.1.3. Ornamentation: striate

4.1.1.3.1. Size large → *Dioscorea pohlii*

4.1.2. Shape: spheroidal

4.1.2.1. Ornamentation: croton pattern

4.1.2.1.1. Size large → *Astraea lobata*

4.1.2.2. Ornamentation: microreticulate

4.1.2.2.1. Size medium → *Bulbostylis paraensis*

Rhynchospora seccoi

Rhynchospora tenuis

Scleria cyperina

Scleria verticillata

Dioscorea glandulosa

4.1.2.3. Ornamentation: punctate

4.1.2.3.1. Size large → *Heliconia adeliana*

4.1.2.4. Ornamentation: reticulate

4.1.2.4.1. Size large → *Vellozia sp.*

Vellozia sp.

4.1.2.5. Ornamentation: reticulate heterobrochate

4.1.2.5.1. Size large → *Aechmea mertensii*

4.1.2.5.2. Size medium → *Aechmea castelnavii*

4.2. Aperture: inaperturate with pseudoapertures

4.2.1. Shape: elliptical

4.2.1.1. Ornamentation: microreticulate

4.2.1.1.1. Size medium → *Cyperus haspan*

4.2.2. Shape: spheroidal

4.2.2.1. Ornamentation: microreticulate

4.2.2.1.1. Size medium → *Cyperus aggregatus*

Cyperus amabilis

5. Pollen grains mesocolpi

5.1. Aperture: 3-mesocolpi fused in pairs

5.1.1. Shape: prolate spheroidal

5.1.1.1. Ornamentation: reticulate

5.1.1.1.1. Size large → *Passiflora glandulosa*

Passiflora tholozanii

6. Pollen grains porate

6.1. Aperture: di(tri)porate

6.1.1. Shape: oblate spheroidal

6.1.1.1. Ornamentation: reticulate to rugulate

6.1.1.1.1. Size large → *Aechmea bromeliifolia*

6.2. Aperture: monoporate

6.2.1. Shape: spheroidal

6.2.1.1. Ornamentation: microreticulate

6.2.1.1.1. Size large → *Paspalum carajasense*

6.2.1.1.2. Size medium → *Axonopus carajasensis*

Axonopus longispicus

Eragrostis maypurensis

Eragrostis rufescens

Hildaea breviscrobs

Ichnanthus calvescens

Isachne polygonoides

Mesosetum cayennense

Mnesithea aurita

Otachyrium versicolor

Paspalum carinatum

Paspalum virgatum

Rhytachne gonzalezii

Trichanthecium sp.

6.2.1.2. Ornamentation: microreticulate-microrugulate

6.2.1.2.1. Size medium → *Axonopus capillaris*

6.2.1.3. Ornamentation: microreticulate-reticulate

6.2.1.3.1. Size medium → *Sporobolus multiramosus*

Trichanthecium parvifolium

Trichanthecium polycomum

6.3. Aperture: pantoporate

6.3.1. Shape: prolate spheroidal

6.3.1.1. Ornamentation: psilate and microreticulate

6.3.1.1.1. Size very large → *Mandevilla hirsuta*

6.3.1.2. Ornamentation: reticulate

6.3.1.2.1. Size medium → *Mandevilla tenuifolia*

6.3.2. Shape: quadrangular

6.3.2.1. Ornamentation: microreticulate

6.3.2.1.1. Size large → *Banisteriopsis appressa*

6.3.3. Shape: spheroidal

6.3.3.1. Ornamentation: echinate and microreticulate

6.3.3.1.1. Size large → *Ipomoea asplundii*

Ipomoea carajasensis

Ipomoea cavalcantei

6.3.3.1.2. Size large to very large → *Ipomoea cavalcantei*

Ipomoea decora

Ipomoea procumbens

Ipomoea setifera

Turbina cordata

6.3.3.1.3. Size very large → *Ipomoea goyazensis*

Ipomoea marabaensis

6.3.3.2. Ornamentation: microreticulate

6.3.3.2.1. Size medium → *Spermacoce sp.*

Spermacoce sp.

6.3.3.3. Ornamentation: puntate with microechinae

6.3.3.3.1. Size large → *Borreria latifolia*

6.3.3.3.2. Size medium → *Borreria alata*

Borreria elaiosulcata

6.3.3.3.3. Size small → *Borreria paraensis*

6.3.4. Shape: spheroidal to hexangular

6.3.4.1. Ornamentation: microreticulate

6.3.4.1.1. Size large → *Banisteriopsis sp.*

6.3.5. Shape: spheroidal to quadrangular

6.3.5.1. Ornamentation: microreticulate

6.3.5.1.1. Size large → *Banisteriopsis malifolia*

Diplopterys pubipetala

6.4. Aperture: pantoporate

6.4.1. Shape: spheroidal

6.4.1.1. Ornamentation: psilate to punctate

6.4.1.1.1. Size gigantic → *Costus scaber*

6.4.1.1.2. Size very large → *Chamaecostus acualis*

Chamaecostus lanceolatus

7. Pollen grains spiral

7.1. Aperture: spiraperturate

7.1.1. Shape: spheroidal

7.1.1.1. Ornamentation: microechinate and microreticulate

7.1.1.1.1. Size medium → *Eriocaulon aff.*

7.1.1.1.2. Size small → *Eriocaulon setaceum*

Paepalanthus aff.

Syngonanthus caulescens

Syngonanthus discretifolius

Syngonanthus heteropeplus

Syngonanthus sp.

8. Pollen grains sulcate

8.1. Aperture: monosulcate

8.1.1. Shape: prolate

8.1.1.1. Ornamentation: scabrate to echinate

8.1.1.1.1. Size large → *Socratea exorrhiza*

8.1.2. Shape: prolate spheroidal

8.1.2.1. Ornamentation: reticulate heterobrochate

8.1.2.1.1. Size medium → *Pitcairnia lanuginosa*

8.1.3. Shape: prolate to perprolate

8.1.3.1. Ornamentation: microreticulate

8.1.3.1.1. Size large → *Attalea maripa*

8.1.4. Shape: subprolate to prolate

8.1.4.1. Ornamentation: reticulate heterobrochate

8.1.4.1.1. Size medium → *Dyckia duckei*

8.2. Aperture: monosulcate

8.2.1. Shape: perprolate

8.2.1.1. Ornamentation: microreticulate

8.2.1.1.1. Size large → *Euterpe oleraceae*

8.3. Aperture: trichotomosulcate

8.3.1. Shape: oblate spheroidal

8.3.1.1. Ornamentation: microreticulate

8.3.1.1.1. Size large → *Acrocomia aculeata*

8.4. Aperture: trichotomosulcate

8.4.1. Shape: perprolate

8.4.1.1. Ornamentation: microreticulate

8.4.1.1.1. Size medium → *Oenocarpus distichus*

8.5. Aperture: zonasulcate

8.5.1. Shape: subprolate

8.5.1.1. Ornamentation: rugulate

8.5.1.1.1. Size large → *Onychopetalum amazonicum*
